# A prokaryotic-like cardiolipin synthase is essential to maintain mitochondrial function, lipid homeostasis, and survival of the blood stage malaria parasite *Plasmodium falciparum*

**DOI:** 10.1016/j.jbc.2026.113188

**Published:** 2026-05-26

**Authors:** Md Muzahidul Islam, Md Omair Anwar, Yoshiki Yamaryo-Botté, Pragyan P. Rath, Madiha Abbas, Vandana Thakur, Sumit Rathore, Cyrille Y. Botté, Asif Mohmmed

**Affiliations:** 1 International Centre for Genetic Engineering and Biotechnology, New Delhi, India; 2 ApicoLipid Team, Institute for Advanced Biosciences, CNRS UMR5309, Université Grenoble Alpes, INSERM U1209, Grenoble, France; 3 All India Institute of Medical Sciences, New Delhi, India

**Keywords:** membrane biogenesis, mitochondrial ETC, cardiolipin, organelle homeostasis, Plasmodium, malaria

## Abstract

Mitochondrion is essential for the propagation of the malaria parasites in the human host. The structure, biogenesis, and function of mitochondria depend on the synthesis of a key mitochondrial membrane-specific phospholipid, cardiolipin (CL), which is typically synthesized by cardiolipin synthase (CLS). Unlike most eukaryotic cell models, the synthesis of CL in the malaria parasite remains unresolved. Here, we show that *Plasmodium falciparum* harbors a prokaryotic-like CLS (*Pf*CLS), which is functional in the parasite mitochondrion. Inducible knockdown of *Pf*CLS disrupts parasite development and kills the parasites. Ablation of *Pf*CLS and a reduction in levels of CL affect the assembly of mitochondrial respiratory super-complexes. In addition, *Pf*CLS-iKD parasites exhibited hypersensitivity to antimalarial drugs targeting the mETC, such as Atovaquone. Lipidomic analyses revealed loss of CL content and widespread perturbations in phospholipid homeostasis in the parasites. Quantitative metabolomics shows disruption of the TCA cycle and amino acid levels. Overall, these results ascertain that *Pf*CLS is essential for asexual blood stages of the parasite, playing a critical role at the juncture of mitochondrial membrane biogenesis, mitochondrial function, and phospholipid synthesis/homeostasis in the parasite.

Despite remarkable developments in the human healthcare system, malaria remains a global burden, mainly in the tropical and subtropical regions, causing substantial morbidity and mortality. Globally, malaria resulted in ∼ 263 million cases leading to ∼ 597,000 mortalities in the year 2023, the majority of which deaths are among children aged below 5 years (1). Among five different *Plasmodium* spp., *Plasmodium falciparum* causes the most fatal form of disease, cerebral malaria. Considerable improvement has been made to control malaria in the last decade, due to the availability and effectiveness of artemisinin combination therapy (ACT) (2, 3). Importantly, there is a rapid and massive emergence of parasites resistant to the most used anti-malarial, including the current frontline drug artemisinin, as well as an important threat of further spreading of infected mosquito vectors and thus the disease due to global warming (4, 5, 6, 7, 8). Thus, there is an urgent need to identify new drug targets and develop new antimalarials.

The malaria parasites, or *Plasmodium* spp., are part of the Apicomplexa phylum, which includes unicellular eukaryotes, or protists, for which most organisms are obligate intracellular parasites of eukaryotic organisms and cells. Beyond the endomembrane system typical of eukaryotic cells (nuclear envelope, ER, Golgi), the parasites possess two essential semi-autonomous organelles present as a single copy in each parasite: the mitochondrion and the apicoplast, both organelles are of prokaryotic origin and have been acquired by different endosymbiotic events (9, 10, 11, 12). Each organelle harbors essential metabolic pathways for parasite survival (13, 14, 15). Therefore, due to their prokaryotic or plant origin and the divergent nature of the metabolic pathways held in these organisms they represent good drug targets. Identification and targeting those unique and essential metabolic pathways of the parasite is a pre-requisite for development of novel drug candidates. The parasite possesses a single, tubular mitochondrion that remains morphologically simple during its intraerythrocytic stages and lacks a fully functional tricarboxylic acid (TCA) cycle (16, 17). Despite this simplification, the electron transport chain (ETC) remains essential, not for ATP synthesis, but for sustaining mitochondrial membrane potential and enabling dihydroorotate dehydrogenase activity, critical for pyrimidine biosynthesis (18, 19).

Lipid biosynthesis, particularly the phospholipid biosynthesis, represents a critical metabolic pathway of the parasite. These pathways are essentially required for membrane biogenesis and hence indispensable for growth and division of the parasite in the human host, making it an important and promising avenue for finding novel drug targets (20). During its massive intraerythrocytic growth, the parasite produces *de novo* large amounts of membrane to support expansion of its parasite plasma membrane (PPM), the parasitophorous vacuole membrane (PVM) and other membranous structures of the growing parasite. Major phospholipids of *Plasmodium* include phosphatidylcholine (PC), phosphatidylethanolamine (PE), phosphatidylserine (PS), and phosphatidylinositol (PI) (21, 22, 23, 24). Amid the predominant presence of standard phospholipids in *Plasmodium* membranes, another phospholipid, cardiolipin (CL), emerges as a unique mitochondrial lipid with its specialized biophysical characteristics and functions.

Cardiolipin (CL) is a mitochondria-specific glycerophospholipid with four fatty acyl chains and two phosphate groups, which makes it unique from other glycerophospholipids (25). Based on its tendency to form non-bilayer structures CL adopts a hexagonal phase and thus localizes preferentially to sites with high membrane curvature, found at mitochondrial cristae (26, 27) and contact sites of inner and outer mitochondrial membranes (28). It plays a crucial role in the organization of membrane protein complexes, regulation of mitochondrial dynamics, and maintenance of membrane potential (29). CL tightly associates with respiratory complexes and is required for the assembly and stabilization of respiratory super complexes of ETC components that facilitate efficient electron transfer and reduce reactive oxygen species (ROS) generation (30, 31). In some organisms CL also helps the oligomerization of Complex V, ATP synthase (26). Recently, depletion of CL has been also shown to effect on cell's energy metabolism by impairing the tricarboxylic acid (TCA) in *Saccharomyces cerevisiae* (32).

The biosynthesis of CL occurs on the matrix side of the inner mitochondrial membrane in four steps *via* the intermediate phosphatidic acid, CDP-diacylglycerol, phosphatidyl glycerophosphate and phosphatidylglycerol. In most prokaryotes, CL is produced using two molecules of phosphatidylglycerol (PG) as substrates by prokaryotic-type CL synthases (33), whereas in most eukaryotes, phosphatidylglycerol reacts with phospholipid cytidine diphosphate diacylglycerol (CDP-DAG) to form CL by eukaryotic-type CL synthases (34, 35). The catalytic sites of almost all prokaryotic Cls contain phospholipase D (PLD)-like motifs, whereas those of eukaryotic Cls contain motifs of enzymes belonging to the CDP-alcohol phosphatidyl transferase family, such as phosphatidyl and phosphotransferases (36, 37). As exceptions, parasitic protists of the phyla Apicomplexa and Euglenozoa, including the human pathogenic parasites *Trypanosoma brucei*, *Trypanosoma cruzi*, and *Leishmania* spp, encode bacterial-type CL synthases (37, 38, 39).

Cardiolipin synthase (CLS) plays a pivotal role in mitochondrial function across diverse organisms; its absence has major physiological effects. Deletion of the CLS gene (crd1Δ) alters mitochondrial DNA integrity, slows the G2/M phase of the cell cycle, and triggers stress signaling pathways in *S. cerevisiae* (40). In *Escherichia coli*, deletion of all three cardiolipin synthase isoenzymes (ClsA, ClsB, ClsC) affects membrane lipid composition, therefore influencing osmosensing and membrane protein localization (41). In parasitic protozoa, CLS is equally critical. For instance, inducible knockdown of CLS in *T. brucei* causes rapid reduction of ATP levels and mitochondrial membrane potential, destabilizes respiratory supercomplexes, and eventually leads to parasite death (42, 43).

In the present study, we have functionally characterized *P. falciparum* cardiolipin synthase, *Pf*CLS (PlasmoDB Gene ID: PF3D7_0609400), to assess its role for blood-stage parasite growth, mitochondrial membrane biogenesis, and lipid homeostasis and linked with parasite energy metabolism. Here, we used HA-*glm*S ribozyme-based conditional gene disruption approach combined with lipidomic, metabolomic and proteomic analysis. *Pf*CLS was localized in the mitochondrion of the parasite, whereas transient knock-down of *Pf*CLS confirmed its essentiality for parasite survival and its role in the development of functional mitochondria. Detailed lipidomic analyses showed that parasites lacking *Pf*CLS displayed fluctuations in phospholipid levels, a significant reduction of mitochondrial signature lipid CL as well PE and PC. In addition, downregulation of *Pf*CLS and in turn reduced levels of CL, effects the assembly and stabilization of mitochondrial respiratory super-complex especially Complex III and Complex IV. *Pf*CLS knockdown parasites were also found to be hypersensitivity to atovaquone and DSM265, which are antimalarial drugs targeting cytochrome complex (Complex III) in mETC and dihydroorotate dehydrogenase, respectively. In addition, there was a significant metabolic shift in response to *Pf*CLS-iKD, characterized by suppressed oxidative metabolism and enhanced amino acid catabolism. Overall, these results suggest a specific and essential role of *Pf*CLS in mitochondrial membrane biogenesis, maintaining lipid homeostasis, parasite energy metabolism as well as overall parasite survival.

## Results

### Identification and sequence analysis of a bacterial type cardiolipin synthase homologue in *P. falciparum*, *Pf*CLS

A sequence search in the *P. falciparum* genome database (PlasmoDB) identified a putative Cardiolipin Synthase (CLS) protein encoded by a gene PF3D7_0609400. The *P. falciparum* CLS (*Pf*CLS) is a 603 amino acid long protein with a predicted molecular mass of 72 kDa and contains two bacterial type signature Phospholipase D (PLD) domains (98–203 and 355–523 amino acid) (Fig. S1*A*). A sequence analysis and phylogenetic search revealed that PfCLS encodes a putative bacterial-type CLS, as it contains two highly conserved H(X)K (X)4D motifs found in the active sites of two PLD domains (Fig. S1*B*), which is similar to bacterial-type CLS. Homologues of *Pf*CLS are also identified from *Plasmodium berghei* (PBANKA_0108000), *Plasmodium chabudi chabudi* (PCHAS_0108600), *Plasmodium vivax* strain (PVP01_1139500), *Plasmodium knowlesi* (PKNH_1140800) *Plasmodium yoelii yoelii* 17X (PY17X_0109600) and *Plasmodium cynomolgi* (PcyM_1142300) using the genome database. An alignment of the predicted proteins sequences of these genes showed that *Pf*CLS is highly conserved among these *Plasmodium* species and other apicomplexans (Fig. S1*B*). Phylogenetic analysis of homologs of CLS from different eukaryotes, bacteria, and protists was performed, and a highly supported monophyletic clade was formed; within this clade, homologs from Apicomplexan and bacteria were clustered together and form groups corresponding to their source lineages (Fig. S1*C*), whereas CLS from other eukaryotes were grouped into separate clad. Taken together, these results suggest that *P. falciparum* encodes a cardiolipin synthase homolog with characteristic features of bacterial-type CLS enzymes, including two PLD domains. The Cardiolipin (CL) synthesis pathway diverges between eukaryotes and prokaryotes at the final step, the eukaryotic CLS uses PG and CDP-DAG to produce CL with release of CMP, whereas prokaryotic CLS utilizes two PG, releasing glycerol (Fig. S1*D*).

The bacterial and eukaryotic type CLS are shown to be significantly different in terms of functionality and protein structure. The catalytic sites of almost all prokaryotic CLS contain phospholipase D (PLD)-like motifs, whereas those of eukaryotic CLS contain motifs of enzymes belonging to the CDP-alcohol phosphatidyltransferase (CAP) family, such as phosphatidyl and phosphotransferases (36, 37, 44). In prokaryotes, CLS (*E. coli* ClsA) adopts a phospholipase D-type α/β hydrolase-like fold with conserved HKD motifs embedded within a compact, membrane-anchored structure of six to seven transmembrane helices. However, in higher eukaryotes CLS (Human CRLS1) belongs to the CDP-alcohol phosphatidyltransferase family, featuring a distinct membrane-anchored fold with the conserved HXGH motif located in a cytosolic loop forming a catalytic cleft that accommodates CDP-DAG and PG. Structural modelling analysis showed that *P. falciparum* CLS (*Pf*CLS) adopts a distinct predicted fold that shows limited sequence homology to canonical bacterial or eukaryotic CLS enzymes. However, it intriguingly contains two bacterial type non-canonical HKD motifs which is suggestive which is suggestive of a potentially divergent catalytic mechanism, possibly involving a ‘head-group exchange reaction’, although this requires further experimental validation. The tertiary structure shows an extended and atypical topology, which may reflect adaptation to mitochondrial membrane architecture and lipid composition in the parasite (Supplementary data, Fig. S2). Additionally, the recombinant *Pf*CLS also showed concentration-dependent phospholipase D activity (Supplementary data, Fig. S3). These differences between bacterial-type CLS in the parasite as compared to eukaryotic type, make the *Pf*CLS a divergent and thus potential candidate for drug targeting.

### Endogenous tagging of *pfcls* gene reveal a mitochondrial localization of the fusion protein PfCLS

**Figure 1:**
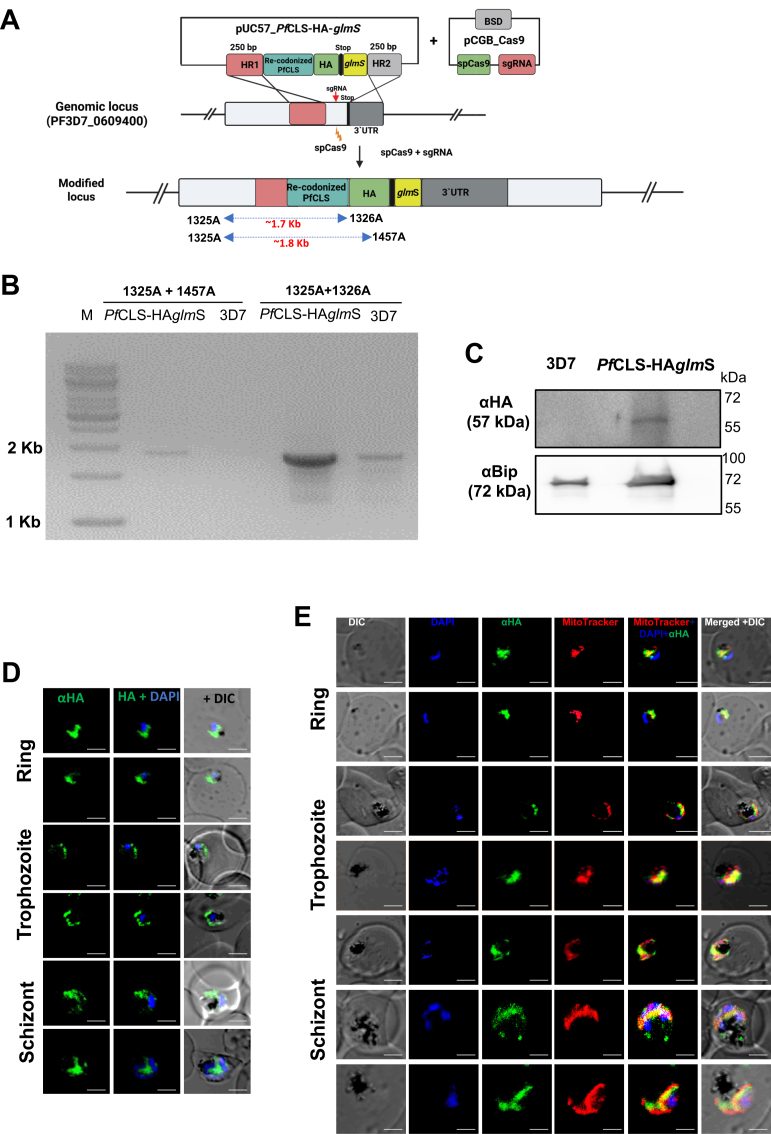
**Generation of transgenic parasites expressing *Pf*CLS-HA-*glm*S using CRISPR-Cas9 strategy, subcellular localization and transient knockdown of *Pf*CLS in the parasite.***A*, schematic representation of the CRISPR-Cas9–mediated gene-editing strategy targeting the *pfcls* locus. The diagram shows the plasmid construct and the expected homologous recombination event directed by sgRNA and Cas9 at the *pfcls* locus. The nucleotide sequence of the construct is provided in Supplementary Data. *B*, PCR-based analysis of transgenic parasites and wild-type 3D7 parasite line to confirm the integration of the HA-*glm*S tag at the *pfcls* gene locus. 1325A, 1326A, and 1475A were the primers used, and their location and expected size of amplicon is shown in the above schematic. *C*, Western blot analysis of transgenic and wild-type 3D7 parasite lysates using anti-HA antibody. A band of 57 kDa was detected in the lysate of transgenic parasites (*Pf*CLS-HA*glm*S) but not in wild-type 3D7 parasites. The *lower panel* shows the blot run in parallel and probed with anti-BiP antibodies as loading controls. *D*, fluorescence microscopy images of transgenic parasites at different developmental stages, immunostained with an anti-HA antibody, demonstrating the expression of the *Pf*CLS–HA fusion protein throughout the asexual stages of the parasite. The nuclei were counterstained with DAPI (*blue*). Scale bar, 2 μm. *E*, confocal microscopy images of transgenic parasites immunostained with an anti-HA antibody and labelled with the mitochondrial dye MitoTracker *Red* across different intraerythrocytic stages of *Plasmodium falciparum*. *Pf*CLS localization was observed to colocalize with MitoTracker-labeled mitochondria throughout the asexual cycle. Parasite nuclei were counterstained with DAPI. Scale bar, 2 μm. DIC, differential interference contrast.

To decipher the localization, as well as to investigate the functional role of *Pf*CLS in the parasite at the asexual blood stages, we generated a transgenic parasite line in which the C-terminus of the endogenous *pfcls* gene was tagged with the HA-*glmS* ribozyme tag. To this end, we utilized the CRISPR-Cas9 strategy for carrying out marker-free integration of the HA-*glmS* tag at the C-terminus of *pfcls* locus in the parasite genome by double crossover homologous recombination (Fig. 1*A*). This transgenic *Pf*CLS-HA*glmS* transgenic parasite line was used to assess the localization of the *Pf*CLS, as well as to investigate its functional significance in the parasite by *glmS* ribozyme-based inducible knock-down strategy. The integration of the HA-*glmS* tag at the C-terminus of *Pfcls* locus was confirmed by PCR-based analysis using genomic DNA isolated from a clonally selected transgenic parasite population (Fig. 1*B*). The amplicon with respect to the integrant-specific primers combination, was obtained only in the case of the transgenic parasites and not in the wild-type 3D7 parasite line, confirming the integration of HA-*glmS* tag at the C-terminus of the native *pfcls* gene locus. The expression of fusion protein was confirmed by western blotting using an antibody against the HA tag; the detection band at the expected size of fusion protein, was obtained only in the case of transgenic parasites and not in the wild-type parasite lysate (Fig. 1*C*).

To study the physiological localization of the protein, immunofluorescence assay was performed using anti-HA antibody, with parasite at different developmental stages of asexual cycle; the signals for HA staining were obtained across the blood stages, signifying the expression of *Pf*CLS at all developmental stages of the intraerythrocytic life cycle (Fig. 1*D*). Moreover, the staining pattern indicated the localization of protein in a cellular organelle that showed characteristic shape, structure, and division pattern of the parasite mitochondrion during the asexual blood-stage cycle. In the early stages of the parasite, the mitochondrion is present as a small structure close to the nucleus. In the trophozoite-stage parasites, the mitochondrion is present initially as an elongated tubular structure and as a branched structure in the later stages. The CLS is known to be localized in the mitochondria across different living systems (39, 45, 46). To determine the localization of CLS in the parasite mitochondria, we performed the colocalization study using MitoTracker dye, which labels the mitochondria of the parasite. As expected, the MitoTracker and HA staining were found to be overlapping, confirming the localization of *Pf*CLS in the parasite mitochondria across the blood stage of the parasite (Fig. 1*E*).

### Downregulation of PfCLS inhibits parasite growth and disrupts intraerythrocytic parasite cycle

**Figure 2:**
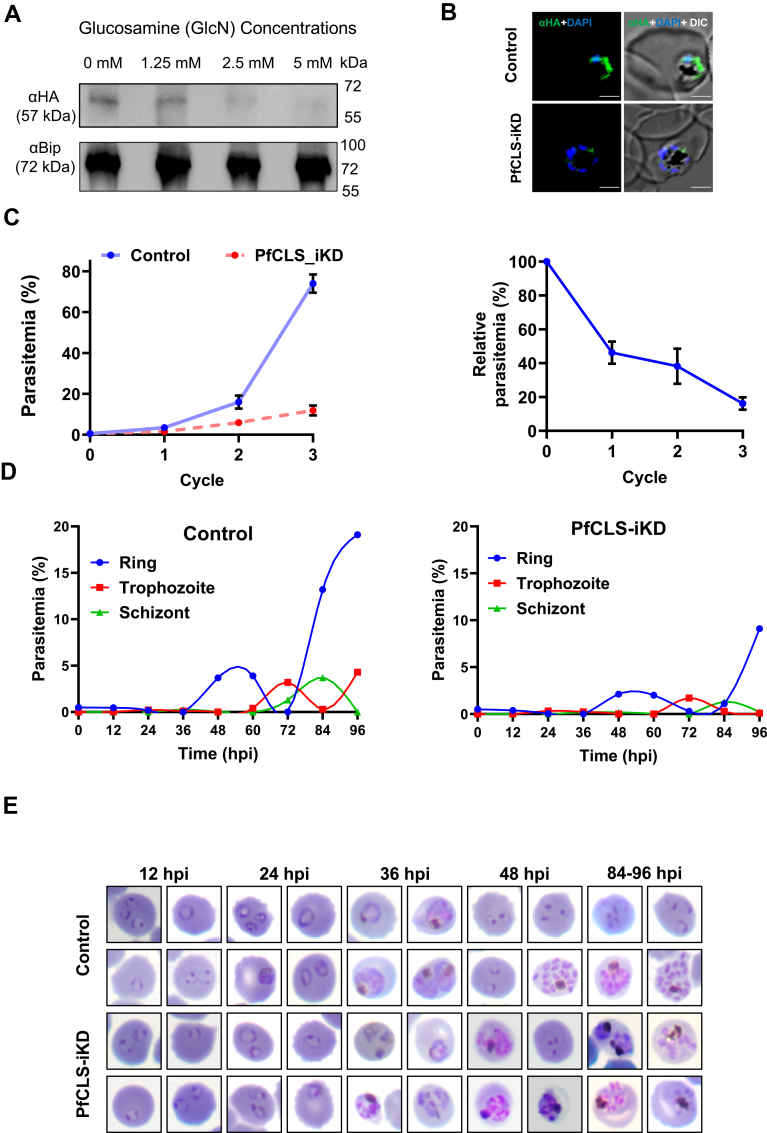
**Inducible gene disruption confirms the essential role of *Pf*CLS in parasite growth and development during the asexual life cycle.***A*, immunoblot analysis using anti-HA antibody showing a dose-dependent reduction in the levels of *Pf*CLS-HA fusion protein in transgenic parasites grown in the presence of different concentrations of glucosamine. A blot run in parallel and probed with anti-BiP antibodies was used as control. *B*, representative fluorescent microscopy images of the transgenic parasites grown in the presence of glucosamine (iKD) or without (control), stained with anti-HA antibody, showing reduction in the levels of *Pf*CLS-HA fusion protein labelling. Scale bar, 2 μm. *C*, graph showing growth over four erythrocytic cycles for *Pf*CLS-HA-*glm*S parasites treated with glucosamine (iKD) in compared to untreated control. The normalized cumulative parasitemia was determined by counting total number of parasites at 48, 96, 144 and 192 hpi. The relative parasitemia values are also shown for *Pf*CLS-iKD culture as compared to control. All analyses were performed in triplicate (*n* = 3); error bars indicate standard deviations. *D*, parasite stage composition of transgenic parasites grown in the presence or absence of glucosamine (*Pf*CLS-iKD and Control, respectively), at different time points (0–96 hpi). *E*, representative images of Giemsa-stained transgenic parasites for control and *Pf*CLS-iKD sets, at different time points (0–48 hpi), showing effects of *Pf*CLS downregulation on parasite morphology.

To investigate the functional role of *Pf*CLS in the parasite growth and development at the asexual blood stage of parasite, we utilized the transgenic *Pf*CLS-HA-*glmS* line for the *glmS* ribozyme mediated transient knock-down of *Pf*CLS. The transient knockdown of *Pf*CLS were assessed in the lysate of the parasite grown in presence of glucosamine (+GlcN) in comparison to the control set (-GlcN), by western blotting using anti-HA antibodies, which shows glucosamine concentration dependent reduction in *Pf*CLS in the parasites; ∼70% reduction in the protein level was observed at 2.5 mM GlcN concentration (Fig. 2*A*). All further experiments were conducted at this concentration of glucosamine to avoid any GlcN-mediated toxicity. The downregulation *Pf*CLS were also confirmed by immuno-staining using anti-HA antibody. In the control set, bright HA staining was observed, while very weak fluorescence was observed in the GlcN treated parasite, signifying the decrease in level of *Pf*CLS-HA fusion protein (Fig. 2*B*).

To decipher the essentiality of *Pf*CLS for parasite growth, we determined the parasitemia at different time point (48 h, 96 h,144 h, and 192 h) after glucosamine treatment (*Pf*CLS-iKD and control set, respectively). The GlcN-treated parasite showed ∼85% growth inhibition in comparison to the control set, signifying the essentiality of *Pf*CLS for the parasite growth at the asexual blood stage (Fig. 2*C*). However, the wild-type 3D7 parasite line showed no significant inhibition in growth upon GlcN treatment (Fig. S4).

To further investigate the effect of *Pf*CLS knockdown on parasite development and morphology, the parasite stage profiles were determined using Giemsa-stained smears made at different time points of the intraerythrocytic life cycle. In the control set, ring stage parasite grows into trophozoite and then schizonts, where merozoites are formed, resulting in rupture of RBC. These merozoites invade new RBCs, effectively increasing parasitemia by 5-fold in the subsequent intraerythrocytic life cycle (Fig. 2*D*). The *Pf*CLS-ablated parasites showed no effect in the morphology of the ring and early-trophozoite stage parasite as compared to the control set; however, these parasites were not able to develop into a healthy late-trophozoite stage parasite; a number of parasites at this stage were found to be stressed, having abnormal darkly stained small aberrant morphology (Fig. 2*D*). These morphological effects were also visible in schizont stage parasites, where a number of aberrant schizonts were observed without any segmentation at 48 h (Fig. 2*E*). Majority of these stressed parasites were unable to form progeny merozoites, resulting in a reduction in parasitemia in the subsequent cycles in comparison to the control set. Overall, these data indicated that the *Pf*CLS ablation caused severe phenotype at the late trophozoite and schizont stages resulting in growth inhibition.

### PfCLS is crucial for mitochondrial morphogenesis

**Figure 3:**
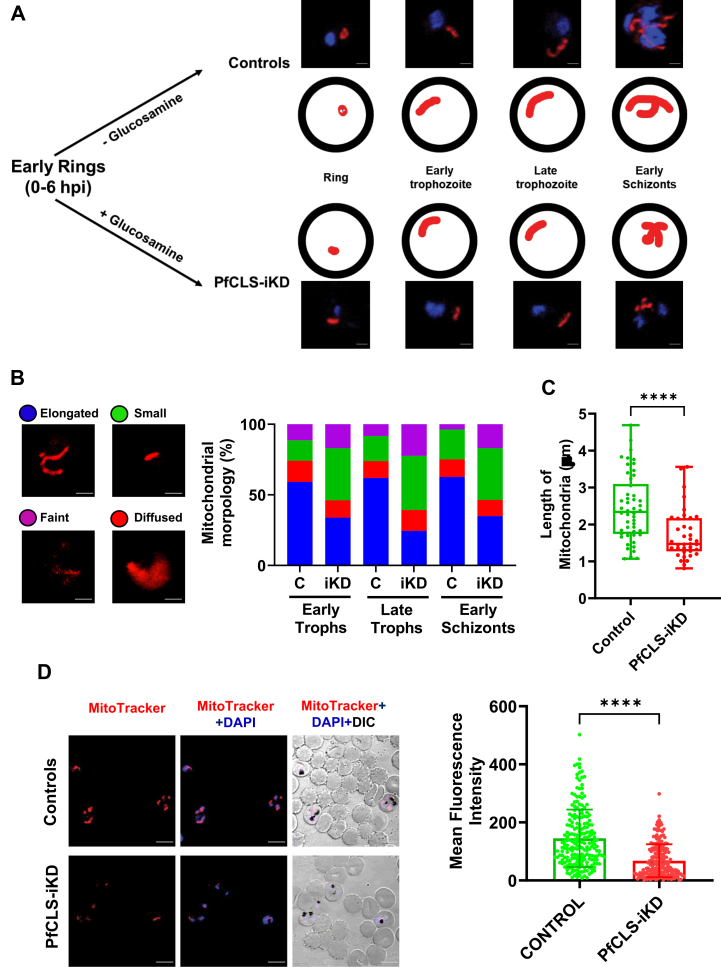
***Pf*CLS plays a critical role in mitochondrial development and segregation in the parasite.***A*, mitochondrial morphology was analyzed in MitoTracker-stained parasites in the *Pf*CLS-iKD set compared to the control set at different time points. Until the early trophozoite stage, mitochondrion was observed to be a small, elongated structure in both control and treated parasites. However, the late trophozoite mitochondria in treated parasites were smaller than those in control parasites. Furthermore, in the schizont stage, mitochondria were well branched in the control parasites, wheras in the treated parasites abnormal mitochondria were observed with small blebbing. The nuclei were stained with DAPI. A Schematic illustration of mitochondrial morphogenesis in the control and *Pf*CLS-iKD sets is shown to present the data. Scale bar, 2 μm. *B*, based on mitochondrial morphology/staining patterns in *Pf*CLS-iKD parasites, four categories were identified: (i) elongated/normal, (ii) small/oval, (iii) faintly stained, and (iv) diffuse without a clear organellar structure. Representative images of each category are shown. The bar graph shows the percentage of parasites displaying each mitochondrial morphology during the early and late trophozoite and early schizont stages in control and iKD parasites. Data represent cumulative counts of at least 100 parasites per stage from two independent experiments, with the graph showing the mean percentage values. Scale bar, 2 μm. *C*, Bar graph comparing mitochondrial length between control and *Pf*CLS-iKD parasites at the late trophozoite stage. Data represent cumulative measurements from three independent experiments (total *n* = 50 for control, *n* = 37 for *Pf*CLS-iKD), with values shown as mean ± SD. Statistical significance was assessed using an unpaired two-tailed Student’s *t* test. *D*, representative fluorescence microscopy images of control and *Pf*CLS-iKD parasites are shown in *left panel*. Labelling of mitochondria with the live fluorescent mitochondrial dye MitoTracker Red was reduced in *Pf*CLS-iKD parasites in compared to that in control parasites. The nuclei were stained with DAPI. The graph on the *right* shows the quantification of mean fluorescence intensity (MFI) of MitoTracker-labelled mitochondria of individual parasites in the control and *Pf*CLS-iKD parasites. Data represent cumulative counts from two independent experiments (total *n* = 208 mitochondria per group). Values are expressed as mean ± SD. Statistical significance for all the experiments was determined using an unpaired, two-tailed Student’s *t* test. Scale bar, 8 μm.

CLS is known to be essential for mitochondrial development due to critical aspect of cardiolipin in mitochondrial membrane. To analyze the effect of *Pf*CLS downregulation on the mitochondria development in the parasite, morphology of mitochondria of live parasites grown in presence or absence of glucosamine (iKD and control sets) were observed under confocal microscope at different stages of intraerythrocytic cycle. In control, mitochondria were observed to be small, elongated structure at ring and early trophozoite stages, growing into an elongated structure at late trophozoite which subsequently gets branched at schizont stages, before dividing into small globular structure in the merozoite. In the CLS ablated parasites (*Pf*CLS-iKD), no marked difference was observed in mitochondrial morphology in ring and early trophozoite stage as compared to control set (Fig. 3*A*). However, at late trophozoite and schizont, significant abnormalities were observed in the morphology of mitochondria. At late trophozoite stage, mitochondria appeared markedly reduced in size in the *Pf*CLS-iKD parasites relative to those observed in control parasites. To better understand the effect of *Pf*CLS-iKD on mitochondrial growth and development, the morphology of mitochondria was categorized into four categories (elongated/branched, small/globular, faintly stained and diffused structure) based on the major mitochondrial morphologies observed in the *Pf*CLS-iKD at the early schizont stage. Majority of parasites in the control set showed the elongated and/or branched mitochondrial morphology during trophozoites and schizont stages, which depicts healthy growing mitochondria; however, in the CLS ablated parasites, small/blebbed mitochondria was detected in majority of the parasites (Fig. 3*B*), average length of mitochondria in these parasites was calculated to be reduced to half as compared to control set (Fig. 3*C*). In addition, significant percentage of parasites also depicted a faint staining of MitoTracker, which could be due to disruption in mitochondrion membrane potential as MitoTracker is a potential base live staining dye. To confirm this observation of faint MitoTracker staining, we quantitated the mean fluorescence intensity of intensity; the average mean MFI was reduced to nearly half in *Pf*CLS-iKD set as compared to the control set, indicating a disruption in mitochondrion membrane potential in absence of *Pf*CLS (Fig. 3*D*). All these results depicted a significant hampering in mitochondrion growth and development at the asexual stage of parasite in absence of *Pf*CLS.

### Depletion of PfCLS disrupts mitochondrial functioning and induce cellular stress in the parasites

**Figure 4:**
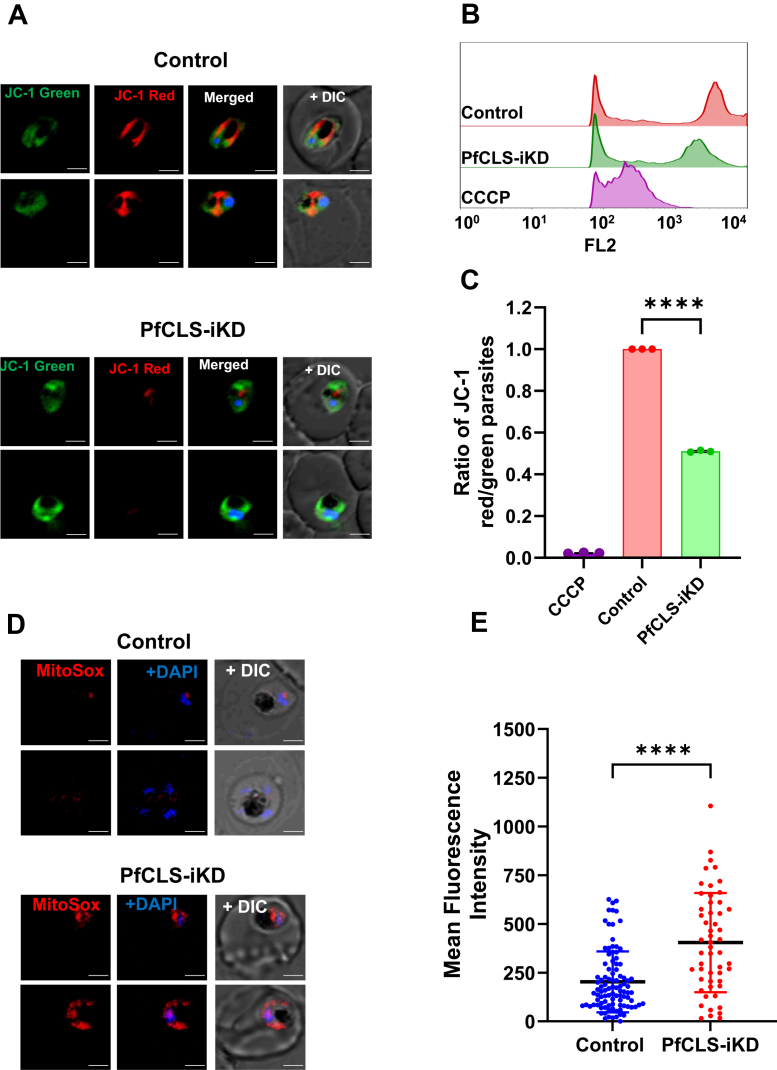
**Effect of downregulation of *Pf*CLS levels on mitochondrial membrane potential and mitochondrial oxidative stress.***A*, representative confocal fluorescence microscopy images of live transgenic parasites of the control and *Pf*CLS-iKD sets stained with JC-1 dye, showing aggregated JC-1 (*red*) in the mitochondria and monomeric JC-1 (*green*) in the cytoplasm. The parasite nuclei were stained with DAPI. Scale bar, 2 μm. *B*, flow cytometry histogram of JC-1-stained transgenic parasites grown in the presence or absence of glucosamine (*Pf*CLS-iKD and Control, respectively) showing a reduction in JC-1 red staining in *Pf*CLS-iKD parasites in comparison to the control, resulting in a reduction in the ratio of JC-1 (*red*)/JC-1 (*green*). Parasites treated with CCCP are used as a positive control. *C*, Bar graph showing ratio of JC-1 *red*/*green* parasite population in control, *Pf*CLS-iKD and CCCP sets. All analyses were done in triplicate, and error bar shows the standard deviation. *D*, *Pf*CLS-HA-tagged transgenic parasites in iKD and control sets, stained with MitoSOX Red Mitochondrial Superoxide Indicator; enhanced *red* fluorescence of MitoSOX is detected in *Pf*CLS-iKD set suggesting higher levels of ROS. Fluorescence microscopic images of parasites from the *Pf*CLS-iKD set showed enhanced *red* fluorescence as compared to the control. Scale bar, 2 μm. *E*, normalized fluorescence intensity of MitoSOX in individual parasites from control and *Pf*CLS-iKD sets. Each point represents an individual parasite, with mean values indicated by horizontal bars. Data represent cumulative measurements from two independent experiments (total *n* = 98 for control, *n* = 53 for iKD). Statistical significance for all the experiments was assessed using an unpaired two-tailed Student’s *t* test (*p* < 0.001).

Cardiolipin is known to be an essential component of mitochondrial membrane across the living system. It maintains the membrane integrity and stabilizes various membrane protein complexes responsible for ion transfer and maintenance of the potential difference across the mitochondrial membrane. In the previous experiment, we observed a decrease in MFI of MitoTracker-stained mitochondria, pointing toward a disruption in membrane potential. To gain mechanistic insight into the severe phenotype observed in the parasite mitochondrion upon CLS downregulation, we checked the mitochondrial membrane potential and the level of reactive oxygen species in the control and GlcN-treated parasites. To assess the mitochondrial membrane potential (Δψ_m_), we used the JC-1 dye-based staining assay. In the cytosol, JC1 gives green fluorescence; while in functional mitochondria, it forms aggregates exhibiting red fluorescence, implying the maintenance of membrane potential. The control set of parasites exhibited red fluorescence of JC-1 aggregates accumulated in the functional mitochondria, while there was significant loss in the red fluorescence in the *Pf*CLS-iKD parasites (Fig. 4, *A* and *B*). The ratio of JC-1 red- and green-stained parasite population was found to be significantly reduced to ∼0.4 in case of *Pf*CLS-iKD parasites, indicating depolarization of mitochondrial membrane in the CLS ablated parasites (Fig. 4*C*).

Further, the ROS production in mitochondria was assessed by utilizing a mitochondrial ROS-specific indicator, MitoSOX, which shows a substantial increase in the normalized mean fluorescent intensity was observed among the parasites in the *Pf*CLS-iKD set, as compared to the control set (Fig. 4*D*). This signifies that *Pf*CLS is associated with keeping a low level of ROS species during mitochondrial-stressed conditions, suggesting a role for *Pf*CLS in maintaining redox homeostasis. (Fig. 4*E*).

### Downregulation of *Pf*CLS disrupts membrane phospholipid synthesis and lipid homeostasis

To investigate the effect of the conditional down-regulation of *Pf*CLS on lipid homeostasis, detailed lipidomic analyses were carried out at trophozoites (30 hpi) and schizonts (40 hpi) stages, when the disruption of *Pf*CLS induces most phenotypical changes of the parasites, and coincides with high levels of *de novo* phospholipid synthesis. We first sought to assess any potential defects in total lipid content and then quantified major lipid classes sustaining parasite survival, free FAs (building blocks of most lipids; FFA), major membrane phospholipids (PLs), diacylglycerols (major lipid precursors, DAG), and triacylglycerols (the main component of lipid storage bodies; TAG).

**Figure 5:**
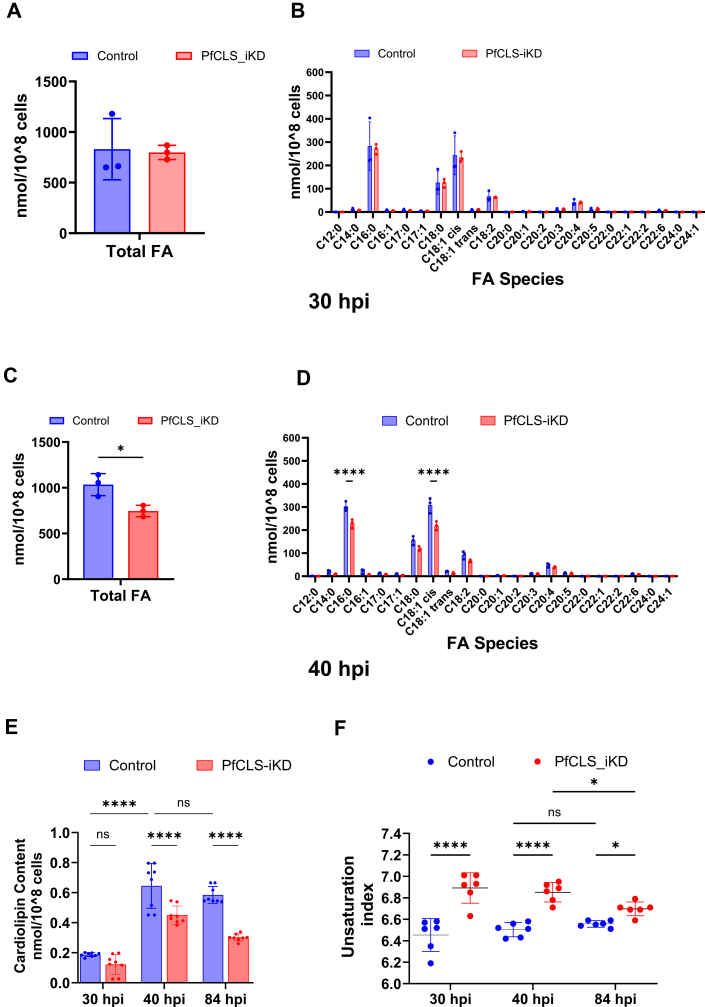
**Lipidomic analyses suggests *Pf*CLS ablation significantly disrupts membrane lipid homeostasis and mitochondrial cardiolipin composition.***A and C*, total fatty acid content (nmol of fatty acids/10^8^ parasites) in control and *Pf*CLS-iKD parasites at 30 hpi (*A*) and 40 hpi (*C*). *B and D*, analysis of fatty acid (FA) composition (nmol lipid/10^8^ parasites) in control and *Pf*CLS-iKD parasites at 30 hpi (*B*) and 40 hpi (*D*), showing no significant differences between the groups. *E*, absolute quantification (nmol/10^8^ parasites) of cardiolipin (CL) content in control and *Pf*CLS-iKD parasites at 30 hpi, 40 hpi, and 84 hpi, showing a significant reduction in CL levels in *Pf*CLS-iKD parasites compared to control. *F*, analysis of the unsaturation index (UI) of CL species in control and *Pf*CLS-iKD parasites at 30 hpi, 40 hpi, and 84 hpi, showing a shift in UI between the two groups. Data represent cumulative measurements from at least three independent experiments. Values are presented as mean ± SD. Statistical significance for all the experiments was assessed using an unpaired two-tailed Student’s *t* test.

During the trophozoite (30 hpi) stage development, the knockdown of *Pf*CLS had no significant effect on the total FA profile of the total parasite lipid content (Fig. 5*A*,). Similarly, knockdown of *Pf*CLS in trophozoite stage (30 hpi) parasites exhibited limited changes, almost no changes in the overall FA profile of the total lipid content of the parasite (Figs. 5*B* and S5*A*). However, some effects in the total lipid FA profile were observed during the schizont stage (40 hpi) (Fig. 5*C*). Further, detailed analysis of the composition of FA molecular species showed perturbations in the levels of FAs, indicating changes in FA homeostasis and fluxes under *Pf*CLS knockdown conditions. The loss of *Pf*CLS resulted in a significant reduction in the relative abundance of C16:0 and C18:1 cis, the most abundant and most essential FA species required for parasite growth (Figs. 5*D* and S5*B*). However, long polyunsaturated fatty acid (PUFA) species remained mostly unaltered.

Interestingly, the phospholipid analysis showed significant changes in *Pf*CLS-iKD set at 30 hpi, even though the total fatty acid levels and profiles remained unchanged. As the CLS catalyze the final step of the reaction cascade to generate mitochondrion-specific phospholipid cardiolipin (CL), depletion of *Pf*CLS would be expected to directly impair *de novo* cardiolipin production. Indeed, at 30 hpi and 40 hpi, a significant reduction of CL content was observed upon *Pf*CLS knockdown (Figs. 5*E* and S6*A*). The Comparative FA analysis of CL composition showed no significant change in abundance at 30 hpi; however, at later stages (40 hpi and 84 hpi), there were significant alterations in FA species composition of Cardiolipin (Fig. S7). We also calculated the CL unsaturation index (UI), which is the weighted average of the double bond number in CLs and the distribution of double bond number across the side chain (Figs. 5*F* and S6*B*). This showed that parasites had maintained a constant UI (mean ∼6.45) in control sets whereas in *Pf*CLS-ikd it significantly increased at 30 hpi, 40 hpi (mean ∼6.85), but a slight decrease was observed at 84 hpi (mean ∼6.735) (Fig. 5*F*). This pattern indicates that *P. falciparum* may initially remodel its cardiolipin composition by increasing unsaturation to adapt to the knockdown stress, which could influence mitochondrial membrane properties, such as enhancing fluidity and function. The fact that the UI remains elevated at 84 h but slightly decreases (6.73) suggests that the adaptation stabilizes over time, possibly due to compensatory mechanisms or metabolic adjustments by modulating the electron transport chain (ETC) or TCA cycle for membrane integrity and energy metabolism.

**Figure 6:**
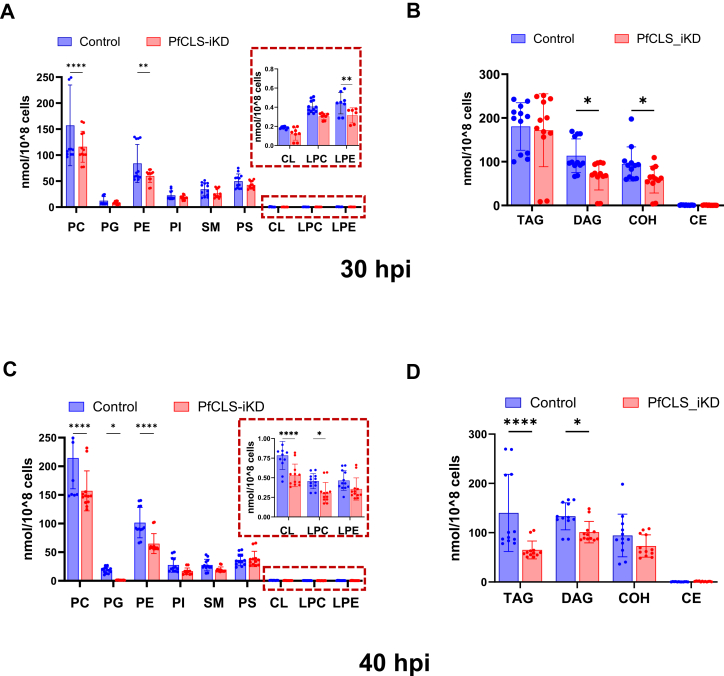
**Lipidomic analyses suggests *Pf*CLS ablation significantly disrupts membrane phospholipid biogenesis.***A and C*, analysis of absolute quantification (nmol lipid/10^8^ parasites) of major phospholipid species (PLs) in control and *Pf*CLS-iKD parasites at 30 hpi (*A*) and 40 hpi (*C*), showing no significant changes in fatty acid composition between control and *Pf*CLS-iKD parasites. Insets show enlarged sections highlighting the relative abundance of CL, LPC, and LPE in CLS-iKD parasites compared to control. *B and D*, Quantification of neutral lipid levels (nmol lipid/10^8^ parasites) in control and *Pf*CLS-iKD parasites at 30 hpi (*B*) and 40 hpi (*D*). Statistical significance for each lipid class in all panels (*A*–*D*) was assessed using an unpaired two-tailed Student’s *t* test, with Holm–Šídák correction for multiple comparisons. All data are presented as mean ± SD from at least three independent experiments (n ≥ 3). Statistical significance is indicated as follows: ∗*p < 0.01, ∗∗p < 0.005, ∗∗∗p < 0.001*. Abbreviations: CL, cardiolipin; LPI, lysophosphatidylinositol; PC, phosphatidylcholine; PE, phosphatidylethanolamine; PG, phosphatidylglycerol; PI, phosphatidylinositol; PS, phosphatidylserine; SM, sphingomyelin.

In addition, the relative abundance of phosphatidylethanolamine (PE) and phosphatidylcholine (PC) were significantly reduced in the knock-down set; there was slight, yet significant reductions in the content of lyso-phosphatidylethanolamine (LPE), while the other PLs, phosphatidylglycerol (PG), phosphatidylserine (PS), phosphatidylinositol (PI) Lyso phosphatidylcholine (LPC) and sphingomyelin (SM), showed only marginal fluctuations at 30hpi (Figs. 6*A* and S8*A*). These effects were enhanced along with the progression of parasite development towards schizonts; the relative abundance of PC, PE, LPE, and LPC were significantly reduced at 40hpi in the iKD set (Figs. 6*C* and S8*C*). The most striking change observed was a major reduction in phosphatidylglycerol (PG) levels at 40 hpi (Figs. 6*C* and S8*C*). PG is usually a low-represented class of membrane PLs that is also the substrate for the essential *de novo* synthesis of the mitochondrial cardiolipin. Analysis of the content of major neutral lipids making the bulk of lipid bodies composition, *i.e.* diacylglycerol (DAG), triacylglycerol (TAG), cholesterol (CHO) and Ceramides (CE) showed that the relative abundance of DAG and CHO was significantly decreased at 30 hpi whereas the one from TAG was normal but at 40 hpi the relative abundance of TAG were significantly decreased (Figs. 6, *B*, *D* and S8, *B*, *D*).

Further, FA contents for phospholipids, PC, PE, PS, PG and PI, analysis at 30 hpi suggested no significant change in abundance in FA species (Fig. S9). However, at the schizont stage (40 hpi), there were significant alterations in the FA species profile of these phospholipids and other lipids (Fig. S9). Significant reductions were evident in the case of following species PC(34:2), PE (34:1), PE(34:2), PE(36:4), PG(32:0), LPC(18:0), LPE(20:4), LBPA(32:0) And PI(36:1)). In contrast, PC(32:1), PE(36:1), PE(36:2), PG(34:1), PG(36:3), LPC(18:1), LPE(18:0), LPE(18:1), LBPA(36:2) and PI (34:1) were significantly increased in abundance upon *Pf*CLS disruption.

### Depletion of PfCLS causes impairment of mitochondrial mtETC and mitochondrial homeostasis

**Figure 7:**
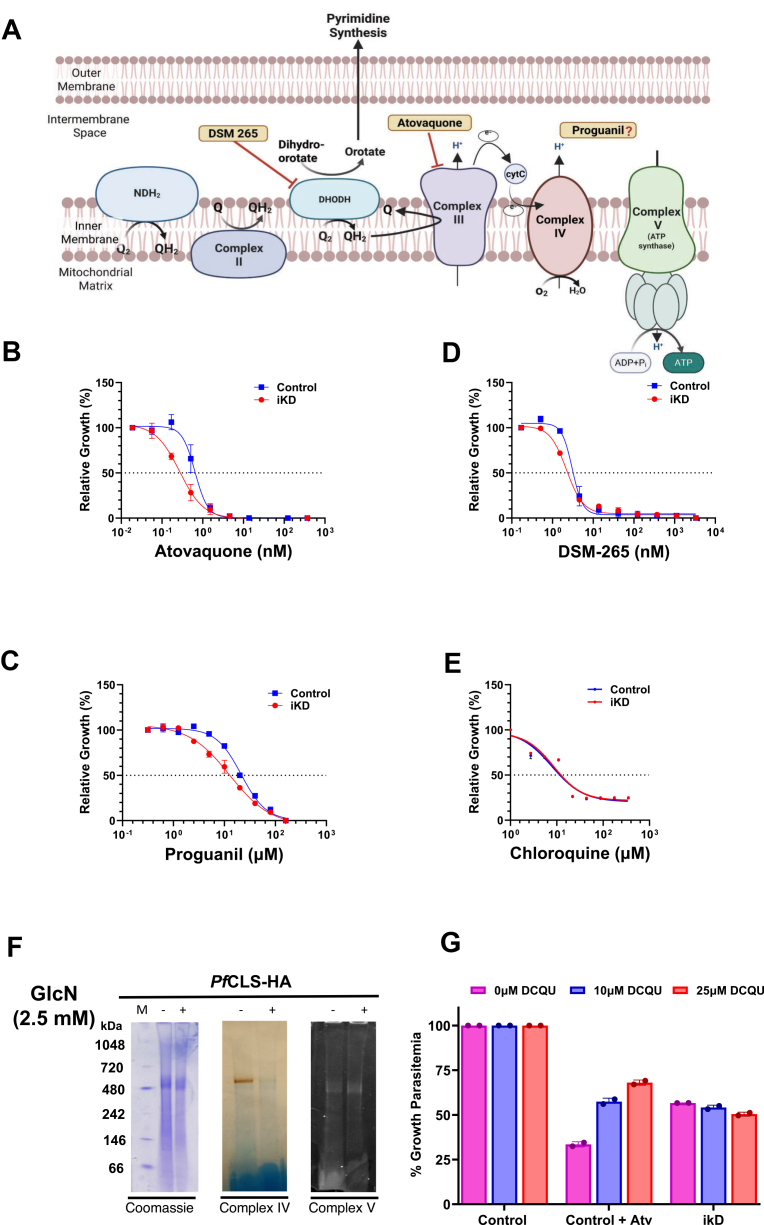
**Depletion of *Pf*CLS hinders mtETC super complex formation and turns parasites hypersensitive to drugs targeting mtETC.***A*, schematic depicting *P. falciparum* respiratory chain complex with mtETC targeting drugs and their expected mode of action. *B–D*, drug susceptibility assays of Control and *Pf*CLS-iKD parasites using Atovaquone (*B*), Proguanil (*C*), DSM265 (*D*) and (*E*) Chloroquine. Parasite growth was assessed by measuring DNA content using SYBR gold when exposed to varying concentrations of drugs for 96 h as compared to control and *Pf*CLS-iKD. Data represent mean ± SD from two independent experiments (n = 2). The accompanying table shows IC_50_ values with 95% confidence intervals for each group. *F*, downregulation of *Pf*CLS leads impairs in respiratory complex IV but not in complex V. Mitochondrial enriched fraction from *Pf*CLS-iKD set and control set at 38 to 40 hpi were separated by high resolution clear-native PAGE, and stained either for complex IV or complex V activity, a parallel gel was stained with Coomassie blue as loading control. Data represent results from one representative experiment, with the repeat experiment shown in the Supplementary Data (Fig. S10). *G*, the artificial electron acceptor decylubiquinone (DCUQ) does not rescue growth of *Pf*CLS-iKD parasites. Control and *Pf*CLS-iKD parasites were grown in presence of various concentrations (0 μM, 10 μM and 25 μM) of DCUQ for two parasites cycles and parasitemia was evaluated as compared to control set. As positive control, parasites were treated with atovaquone were used and rescued with DCUQ. Data represent mean ± SD from two independent experiments.

The mitochondrial electron transport chain (mtETC) of *Plasmodium* has been successfully exploited as a major antimalarial drug target (Fig. 7*A*). In light of the primarily mitochondrial localization of *Pf*CLS, we resolved to investigate the susceptibility of our *PfCLS-depleted* (*Pf*CLS-iKD) parasites to toward the drugs that target components of mtETC: atovaquone, which is known to specifically block the activity of the bc1 complex of the mitochondrial electron transport chain (mtETC), and DSM-265 targeting dihydroorotate dehydrogenase (DHOD). *PfCLS-depleted* parasites exhibited a decrease in IC_50_ values for both the drugs, ∼2.4 fold for atovaquone and ∼1.4 fold for DSM265, in comparison to control parasites (Fig. 7, *B*–*D*). As controls, these parasites did not exhibit hypersensitivity to drugs that do not target the mtETC or related mitochondrial processes, *e.g.* Chloroquine (Fig. 7*E*).

Further, the assembly and activity of the respiratory chain complex IV and the ATP synthase complex V were analyzed in enriched mitochondrial fraction using clear-native PAGE followed by an in-gel complex IV and V enzymatic assays. The signal obtained by Complex IV activity assay showed ∼480 kDa band, which corresponds to the size reported for *Toxoplasma gondii* complex IV (47), supporting the assay’s specificity. Similarly, in-gel activity assay for Complex V, showed expected bands on the native gel (43, 48, 49). The *Pf*CLS depletion showed loss in activity of complex IV; however, the activity of complex V remained unchanged as compared to the control set (Fig. 7*F*; data from an independent replicate experiment are shown in Fig. S10). Taken together, these results show that *Pf*CLS depletion results in a defect in the assembly and activity of a mitochondrial electron transport chain.

A crucial process associated with mtETC is the recycling of ubiquinone for pyrimidine biosynthesis. It has been shown that a ubiquinone analogue, decylubiquinone (DCUQ), can rescue the effects of mtETC targeting drugs. However, *Pf*CLS-iKD parasites could not be rescued by DCUQ as compared to parasites treated with atovaquone (Fig. 7*G*). In addition, the *Pf*CLS-iKD caused increased sensitivity towards Proguanil drug (Fig. 7*C*), which has a broad-ranging effect on mitochondrial functioning/homeostasis through undefined targets (50). These results suggest that the *Pf*CLS-iKD not only results in destabilization of the mtETC complex effecting its function, but also affects the overall mitochondrial homeostasis.

### TCA metabolic profiling upon knockdown of PfCLS

**Figure 8:**
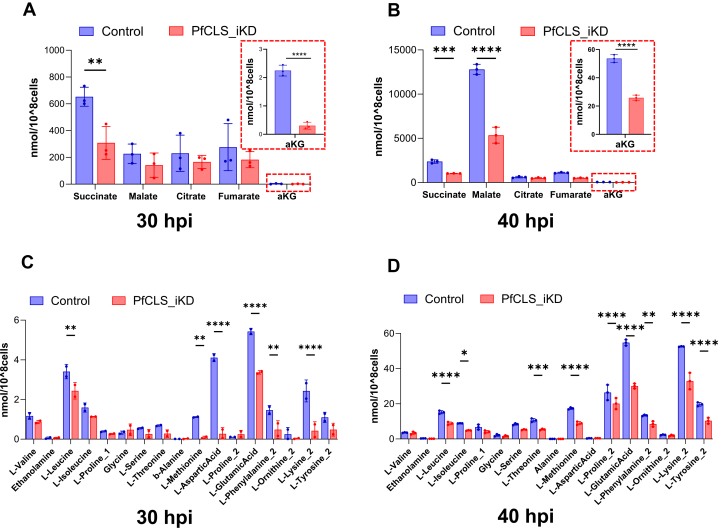
**Depletion of *PfCLS* is associated with disruption in TCA cycle and amino-acids pool/synthesis.** Targeted metabolomics analysis of control and *Pf*CLS-iKD parasite cultures, harvested at 30 hpi and 40 hpi, using liquid chromatography-mass spectrometry (LC-MS). *A, C*, Bar graph showing quantification (nmoles/10ˆ8 cells) of TCA cycle metabolites in the control and *Pf*CLS-iKD sets. Each bar represents the mean concentration ± SD (*n* = 3 biological replicates) for key TCA cycle intermediates: citrate, α-ketoglutarate, succinate, fumarate and malate. Data are presented as mean ± SD from three independent biological replicates (*n* = 3). Statistical significance is indicated as *p* < 0.05 using an unpaired two-tailed Student’s *t* test. *B, D*, Bar graphs showing concentrations of selected amino acids in control and *Pf*CLS-iKD parasites, demonstrating a significant reduction in glutamine, glutamate, alanine, serine, and leucine under knock-down conditions. Data are presented as mean ± SD from three independent biological replicates. Statistical significance is indicated as *p* < 0.05 using an unpaired two-tailed Student’s *t* test.

To gain the further insights of the role *Pf*CLS on cellular energy homeostasis, we investigated the impact of depletion of *Pf*CLS on TCA cycle intermediates and amino acid metabolites by comparing metabolite levels between control and *Pf*CLS-iKD groups. the metabolomic analysis indicate that at 30 hpi the TCA cycle intermediates, succinate and α-ketoglutarate, exhibited statistically significant reduction in *Pf*CLS-iKD as compared to control set (*p* > 0.05) parasites, whereas at 40 hpi revealed significant downregulation of several key TCA cycle intermediates, including malate, succinate, fumarate, and α-ketoglutarate (Fold change ranging from C = 0.42–0.48), in *Pf*CLS-iKD when compared to control set, while Citrate levels remained relatively stable. These results suggests a suppression of oxidative phosphorylation in the *Pf*CLS-iKD (Fig. 8, *A* and *B*). This metabolic shift indicates a potential reduction in mitochondrial function, favouring alternative energy pathways such as glycolysis.

In parallel, amino acid metabolism exhibited profound disruptions, particularly in branched-chain amino acids (BCAAs), with leucine and isoleucine, showing decreased levels (Fig. 8, *C* and *D*). These alterations suggest a potential increase in amino acid catabolism to compensate for impaired ATP production or a decline in protein biosynthesis. Additionally, the downregulation of glutamic acid (FC = 0.55) and methionine (FC = 0.52) implies disturbances in nitrogen metabolism and methylation pathways, which could impact gene regulation and cellular signaling. The significant reduction in tyrosine (FC = 0.52) may potentially influencing cellular stress responses and metabolic homeostasis (Fig. 8, *C* and *D*). One of the most notable drastic depletions observed was β-alanine (FC = 0.17), which is involved in cellular pH maintenance and protection against oxidative stress. This suggests increased metabolic stress in treated cells, potentially reducing their ability to mitigate reactive oxygen species (ROS). Similarly, the downregulation of ethanolamine (FC = 0.49), a precursor for phospholipid biosynthesis, suggests compromised membrane integrity and altered lipid metabolism. Collectively, these results indicate a significant metabolic shift in response to *Pf*CLS-iKD, characterized by suppressed oxidative metabolism, enhanced amino acid catabolism, and increased cellular stress.

## Discussion

Malarial parasites rely heavily on lipid homeostasis and phospholipid synthesis for rapid membrane synthesis during its growth inside host cells. Different lipid and phospholipid biosynthesis pathway are found to be essential in the malaria parasite making these as promising drug targets (22, 51, 52, 53). However, limited information is available on the complex synthesis of phospholipids in parasites. Recent studies have shown a critical association of ER and mitochondria for synthesis of several key membrane phospholipids, including PC, PE and PS (54, 55). In addition to these phospholipids, eukaryotic cells contain a mitochondrial membrane-specific glycerophospholipid, cardiolipin (CL), which plays a critical role in maintaining mitochondrial integrity and function (39, 56); cardiolipin synthase (CLS) catalyzes the synthesis of cardiolipin in the mitochondria.

Here, to further understand lipid homeostasis linked with organelle functional analyses and membrane biosynthesis in the parasite, we explored the role of a putative CLS in *P. falciparum*, *Pf*CLS, during the asexual life cycle of the parasite. Sequence and phylogenetic analyses revealed that *Pf*CLS has characteristic features of bacterial-type CLS, including the presence of the phospholipase domain (PLD) and the characteristic GXSXG motif (39, 57). Indeed, other apicomplexan parasites, including *T. brucei*, *T. cruzi,* and *L.* spp, have been shown to encode bacterial-type CLS (37, 38). Bacterial and eukaryotic type CLS are shown to be significantly different in terms of functionality and protein structure. The structural homology studies have also suggested that *Pf*CLS has bacterial-type phospholipase D-type α/β hydrolase-like fold to facilitate reaction between two PG molecules and synthesis of CLS. As a limitation of the study, we were unable demonstrate the direct PG to CL *in vitro* conversion assay using recombinant *Pf*CLS but lipidomic analyses confirmed that the enzyme is responsible for CL synthesis in the parasite. Importantly, the observed PLD activity assays strongly indicate that *Pf*CLS does not function as a eukaryotic-type enzyme and confirms the prokaryotic-like mechanism of action of the enzyme. The presence of a bacterial-type CLS in the parasite, which is divergent from eukaryotic type, makes *Pf*CLS a tempting drug target.

In *E. coli* and Yeast, CLS knockouts are viable only under specific growth conditions (41, 58), whereas the CLS is shown to be essential for apicomplexan parasite *T. brucei* (39). However, the function and importance of CLS in malaria parasite growth and pathogenesis have not yet been elucidated. Here, the ribozyme system using CRISPR-Cas9-mediated integration was utilized to decipher the role *Pf*CLS (59, 60, 61). The localization of the endogenously expressed PfCLS-fusion protein was confirmed in the parasite mitochondria. Extensive parasite growth inhibition after *Pf*CLS knockdown highlighted the essentiality of this protein for parasite growth and development from trophozoite to schizont stages during the intraerythrocytic stage. The transition from the trophozoite to schizont stage is the most metabolically active phase of the parasite, requiring substantial lipid and membrane synthesis to support organelle biogenesis, daughter merozoite formation, cytokinesis, and karyokinesis. As discussed earlier, Cardiolipin (CL) is one of the key components of mitochondrial membrane, mainly present in curved membranes found at cristae and membrane contact sites within the mitochondria (26, 27), thus it is critical for the maintenance and functioning of the organelle. In this study, *Pf*CLS depletion disrupted mitochondrial development, resulting in the loss of mitochondrial membrane potential, increased mitochondrial oxidative stress, as evidenced by elevated ROS levels, and caused mitochondrial fission/fragmentation, ultimately leading to cell death. Indeed, a genome-wide mutagenesis screen utilizing the transposon system identified *Pf*CLS as an essential protein for the parasite (62). Overall, these results indicate that *Pf*CLS is essential for parasite growth and development at the asexual blood stage, making it a potential drug target.

In view of the importance of membrane biogenesis and lipid homeostasis in the development and multiplication of *P. falciparum*, the enzymes involved in phospholipid synthesis/metabolism are considered central to parasite survival. Indeed, a number of enzymes involved in lipid metabolism are shown to be critical for parasite survival and multiplication in the host cell (57, 63, 64, 65, 66). The membrane PLs are *de novo* synthesized/assembled by the parasite using their precursors (FAs and polar heads), which are scavenged from the host milieu (65, 67). As CLS is one of the key enzymes for mitochondrial membrane biogenesis, mediating the last step in CL synthesis, the role of *Pf*CLS in phospholipid synthesis/homeostasis was analyzed. The lipidomic analysis revealed that, the total fatty acid (FA) phenotype was hampered and significant disruptions were observed within different classes of phospholipids as the parasite growth stage progressed toward trophozoite to schizonts. The cardiolipin (CL) levels, the signature lipid of mitochondrial membrane, showed a major reduction in its levels at the trophozoite stage, which was reflected in the developmental abnormalities in the parasite mitochondria. The shift toward shorter-chain cardiolipin species and an increased unsaturation index (UI) suggests an adaptive remodeling response, potentially aimed at preserving mitochondrial membrane fluidity and function under stress. The slight decrease in the UI at 84 hpi, although still elevated compared to that of the controls, indicates that the parasite may attempt to restore homeostasis through compensatory mechanisms. This stabilization could be linked to metabolic adjustments within the TCA cycle and ETC, allowing the parasite to sustain its bioenergetic demands despite disruptions in cardiolipin biosynthesis.

It has been shown that the ER and mitochondria work together for the synthesis of other membrane phospholipids (54, 68, 69, 70) and organelle stress in any of these organelles also causes disruption of metabolic pathways in the other organelle (54). Similarly, reduction in CL levels and consequent induction of mitochondrial stress after CLS ablation, resulted in a significant reduction in levels of major membrane phospholipids, Phosphatidylcholine (PC), Phosphatidylethanolamine (PE) and DAGs. In addition to activating protein kinase C by acting as a second messenger (71), DAG also serves as a precursor TAG and major PLs syntheis (72). DAGs are utilized in the Kennedy pathway for the synthesis of PC and PE in the ER; therefore, a reduction in DAGs in the ER leads to reduced levels of these PLs. Furthermore, during parasite growth, TAG hydrolysis can rapidly produce FAs to utilize in membrane synthesis and membrane assembly (72, 73). Similarly, the reduction of DAGs in early parasite stages after CLS ablation caused reduced TAGs levels in mature stages. In our earlier study, we showed that the regulation of DAG to TAG conversion, and timely release of FAs for phospholipid synthesis are crucial for membrane generation in late parasite stages (57); disruption of this balance after CLS ablation hampers the normal development of trophozoites into schizonts and future merozoites. One intriguing observation is the downregulation of PG, subsequent to the phenotypic effects of *Pf*CLS-iKD. A plausible mechanistic explanation for this reduction is an early decrease in DAG levels in the *Pf*CLS-iKD parasites, which could subsequently limit phosphatidic acid (PA) availability and thereby impair PG biosynthesis. Overall, this dataset highlights the dynamic nature of *P. falciparum* mitochondrial lipid remodeling in response to *Pf*CLS depletion and mitochondrial stress, emphasizing interdependence.

Mitochondria are typically considered the site of oxidative phosphorylation, which provides energy for eukaryotic cells. However, during asexual blood stages, the *Plasmodium* parasites rely on glycolysis rather than oxidative phosphorylation for ATP synthesis (17, 74, 75, 76, 77). Thus, the sole essential function of the mitochondrial ETC during asexual stages is suggested to be oxidative recycling of ubiquinone or synthesis of precursors required for *de novo* pyrimidine synthesis (13, 16, 78). A functional mtETC is thus needed to pump protons across the MIM to build up a proton electrochemical gradient, with the mitochondrial membrane potential as its major component. *Pf*CLS ablation caused hypersensitivity of *P. falciparum* to mtETC inhibitors, particularly those targeting Complex III, suggesting its vital function in maintaining mtETC integrity. Indeed, previous studies in yeasts indicate that inner mitochondrial membrane lipids like CL and PE are crucial for maintaining ETC activity and efficient ΔΨm production by regulating Complex IV activity and affecting respiratory supercomplex formation between Complex III and IV (79, 80, 81). Further, conditional depletion of *Pf*CLS disrupted the activity of respiratory complex IV, which contains the mitochondrially encoded subunits (Cox I and Cox III), which again confirmed the *Pf*CLS and hence CL is critical in maintaining mtETC integrity. Earlier studies also showed the critical role of CL molecules in maintaining inner membrane integrity and enzymatic activity of mitochondrial respiratory complexes (82, 83).

Targeted metabolomics data provided critical insights into the metabolic consequences of *Pf*CLS depletion in *P. falciparum*, highlighting its role in mitochondrial function and cellular energy homeostasis. The absence of significant metabolic alterations, the observed downregulation of key TCA cycle intermediates indicates a shift away from oxidative phosphorylation, potentially driving the parasite toward glycolysis-dependent energy production. The observed depletion of α-KG upon *Pf*CLS knockdown suggests impaired TCA cycle activity, potentially reducing oxidative phosphorylation and forcing the parasite to rely more on glycolysis for ATP production. Further the concurrent depletion of BCAAs and glutamic acid suggests an increased reliance on amino acid catabolism, likely to mitigate ATP deficits; whereas the reduction in β-alanine and ethanolamine levels points to heightened metabolic stress and compromised membrane integrity.

Overall, these results decipher the effect of CLS ablation and consecutive reduction of CL in the parasite: (i) disruption of critical balance in phospholipid biosynthesis; (ii) disruption of mitochondrial homeostasis; (iii) the mtETC stability is comprised, causing a reduction in membrane potential; and (iv) which ultimately disrupts the TCA cycle and linked metabolic pathways. These results clearly show that the CL synthesis pathway is critical for mitochondrial function, development and parasite survival. As discussed above, *Pf*CLS is a bacterial-type CLS enzyme, this fact, together with the essentiality of *Pf*CLS for the parasite survival, unambiguously validates it as a drug target for malaria.

## Experimental procedures

### Plasmid construction

The plasmid vector pCas9-BSD-sgRNA *(pCGB)* was generated by modifying the plasmids *pL6* and *pUF-*Cas*9* (61). The *BtgZI* adaptor sequence of the modified *pCGB* vector was replaced with the *Pf*CLS specific guide DNA sequence *Pf*CLSgRNA 1651(+), (5′AGTTATTGTGCATATCATCT 3′) using a previously published protocol (42). To produce the donor plasmid DNA template, a design was created that contains: 5′ homology region (DNA sequence ∼250 bp upstream of DSB in *CLS* locus); a recodonized DNA sequence from DSB till *pfcls* codon penultimate to *STOP* codon; 3 × HA sequence fused with *glm*S ribozyme sequence (from *pJRTS-GFP-glmS* plasmid) (84) and 3′ homology region (250 bp long DNA stretch downstream of *Pf*CLS *STOP* codon over its 3′ *UTR*), and was commercially synthesized from Genscript.

### Parasite culture and generation of transgenic parasites

Plasmodium falciparum strain 3D7 was cultured in RPMI media (Invitrogen) supplemented with 0.5% (w/v) Albumax (Invitrogen) 4% hematocrit. Culture was kept static in a gas mixture of 5% carbon dioxide, 5% oxygen and 90% nitrogen at 37 °C (85). Parasite cultures were synchronized by repeated sorbitol treatment following Lambros and Vandenberg (86) and synchronized *P. falciparum* 3D7 ring-stage parasites were transfected with 100 μg of purified plasmid DNA by electroporation (310 V, 950 μF) (87). Transfected parasites were selected over 2.5 mg/ml Blasticidin S (Gibco) and subsequently subjected to on and off drug cycles to get parasites with plasmid integrated into the main genome, and further clonal selection was done to get pure integrant clones by the limiting dilution method. Integration was confirmed by diagnostic PCR using 1457A and 1325A and by IFA, and western blotting using anti-HA antibody.

### Parasite fractionation and Western blotting

To assess the expression of fusion protein in transgenic *P. falciparum* parasite lines, Western blot analysis was carried out. Briefly, Parasites were harvested at trophozoite stage using 0.15% saponin for RBC lysis, the supernatant was collected, and parasites pellet was lysed by freeze-thaw cycle. Laemmli buffer was added to both the fractions boiled, centrifuged, and the supernatant obtained was separated on 12% SDS-PAGE. The proteins were transferred to PVDF membrane (Millipore) and incubated with blocking buffer (5% skim milk in 1 × PBS) followed by incubation with the primary antibody: (monoclonal anti-HA Rat,1:5000 (Roche), rabbit anti-BiP 1:15,000 (54, 57). Blots were washed 3 times with 1 × PBS and subsequently, the blot was incubated for 1 h with appropriate secondary antibody (anti-rabbit, anti-rat or anti-mouse, 1:20,000) conjugated to HRP, and bands were visualised Clarity Western ECL Substrate detection kit (BioRad).

### Immunofluorescence assay (IFA) and fluorescent microscopy

To visualize the mitochondria, the live transgenic parasites were stained with MitoTracker Red CMXRos (Invitrogen) at a final concentration of 50 nM for 30 min at 37 °C. The nuclei of parasites were stained using 4-,6-diamidino-2-phenylindole (DAPI, Sigma) with a final concentration of 5 μg/ml for 15 min at RT/37 °C. Indirect immunofluorescence assays were performed on *P. falciparum* transgenic parasite lines as described earlier (88, 89). Briefly, parasites were fixed in 4% paraformaldehyde, incubated with either rat polyclonal anti-HA (1:1000) or rabbit anti-Bip (1:500) and subsequently washed then incubated with Alexa Flour-488 and- 594 labelled secondary antibody linked goat anti-rabbit, anti-rat or anti-mouse antibody (Invitrogen). The parasite nuclei were stained with DAPI (2 μg/ml). The immuno-stained parasites were viewed using a Nikon A1 confocal laser scanning microscope. Images were captured by Nikon A1 confocal laser scanning microscope and analyzed by Nikon NIS Element software (version 4.1).

### Conditional knockdown and in-vitro growth assays analysis

To assess the effect of downregulation of *Pf*CLS at the protein level, *Pf*CLS-HA*glm*S ring stage parasites (6–12 hpi) were grown in the presence and absence of different concentrations of D-Glucosamine HCl (Sigma-Aldrich) (1.25 mM, 2.5 mM and 5 mM), harvested at late-trophozoite/schizont stages (36–40 hpi), and subjected to immunoblotting. To assess the effect of knockdown of *Pf*CLS on the parasite growth phenotype, tightly synchronized ring stages (06–10 hpi) parasites were treated with 2.5 mM of glucosamine (iKD) or without glucosamine (control) allowed to grow for three consecutive cycles. Parasitemia in Giemsa-stained smears were determined by counting a minimum of 2000 erythrocytes, and comparative growth analysis was performed up to 3 growth cycles (144 hpi). Each assay was performed in triplicate on two different days.

### Mitochondria membrane potential assay and mitochondrial oxidative stress measurement

The mitochondrial membrane potential was assessed by using JC-1 staining dye as described earlier (90, 91). Briefly, infected erythrocytes from parasite cultures in control and *Pf*CLS-iKD sets were incubated with JC-1 (5,50,6,60 -tetrachloro-1,10,3,30 -tetraethylbenzimidazolyl-carbocyanine iodide) at a final concentration of 5 μM, for 30 min at 37 °C. After washing with 1 × PBS, the cells were examined by flow cytometry using FACS Calibur flow cytometer and CellQuestPro software (Becton Dickinson), using green (488 nm) and red (635 nm) filters. Ratio of JC-1(red)/JC-1(green) was calculated to assess the loss of mitochondrial membrane potential. Parasites treated with CCCP (carbonyl cyanide m-chlorophenyl hydrazone) serve as a positive control; the data were analyzed using the FlowJo software to define the population of parasites with red fluorescent signal.

For mitochondrial oxidative stress measurement, *Pf*CLS-HA tagged parasites were grown in the presence and in the absence of glucosamine and stained with MitoSOX Red (Mitochondrial Superoxide Indicator, Thermo Fisher) at a final concentration of 2 μM (90). The parasites were viewed using a Nikon A1 confocal laser scanning microscope as described above. Images were analyzed to determine the mean fluorescence intensities of individual parasites using Nikon-NIS Element software (version 4.1).

### Lipidomic analysis

Lipidomics analysis was performed on four independent cell harvests of the control and iKD sets at two different time point (30 hpi and 40 hpi). Highly synchronous parasite cultures were harvested; the parasites were liberated from the enveloping RBCs by mild saponin treatment with 0.15% w/v saponin. The parasite pellet was subjected to lipid extraction using chloroform and methanol (92) in the presence of butylhydoxytluene, PC (C21:0/C21:0), C13:0 fatty acids and EquiSPLASH Lipidomix lipid standard mix (Avanti). The total lipids were extracted in chloroform/methanol/water (1:3:1, v/v/v) for 1 h at 4 °C, with periodic sonication. Then, the polar and apolar metabolites were separated by phase partitioning by adding chloroform and water to give the ratio of Chloroform/Methanol/Water, 2:1:0.8 (v/v/v). The total lipid extract was dried under nitrogen gas.

For total fatty analysis, total lipid was then derivatized to give fatty acid methyl ester (FAME) using trimethylsulfonium hydroxide (TMSH, Machenery Nagel) for total glycerolipid content. Resultant FAMEs were then analyzed by GC-MS as previously described (66). All FAMEs were identified by comparison of retention time and mass spectra from GC-MS with authentic chemical standards. The concentration of FAMEs was quantified after initial normalization to different internal standards and finally to parasite number.

For phospholipid analysis, Dried lipid extracts were reconstituted in methanol, vortexed vigorously, and incubated at 30 °C for 5 min. One microliter of each sample was injected into an Agilent 1290 Infinity/Infinity II LC system coupled to a 6495C triple quadrupole mass spectrometer. Lipid separation was performed on a ZORBAX Eclipse Plus C18 column (100 × 2.1 mm, 1.8 μm) maintained at 45 °C using a binary gradient of solvent A (water:acetonitrile:isopropanol, 5:3:2 v/v/v, with 10 mM ammonium formate) and solvent B (isopropanol:acetonitrile:water, 90:9:1 v/v/v, with 10 mM ammonium formate). The mass spectrometer operated in dynamic multiple reaction monitoring (DMRM) mode with positive/negative polarity switching and source parameters adapted from Huynh *et al.* (93) with modifications for *P. falciparum* and deuterated lipid standards. Quantification was performed using Agilent MassHunter software, applying calibration curves of known lipid standards, and results were normalized to cell counts.

### Drug susceptibility assay and DCUQ assay

The drug susceptibility assay was assessed as described earlier (94). Briefly, tightly synchronized ring stage parasites culture in both control and glucosamine treated set (control and *Pf*CLS-iKD) were used for drug susceptibility assays targeting the mitochondrial electron transport chain (ETC). The assays were set up in 96-well microtiter plates (Thermo Scientific) with 1% starting parasitemia and 2% hematocrit in a final volume of 200 μl of medium; parasites were incubated with varying concentrations of the following drugs: Proguanil (Sigma), Atovaquone (A7986, Millipore Sigma), DSM-265 (HY-100184, Med Cam Express). All the drugs were dissolved in DMSO and further diluted in culture medium for the assay. In each plate, infected RBCs in the absence of drugs and only treated with DMSO served as positive controls for parasite growth, whereas uninfected RBCs served as negative controls (background). After 96 h of incubation, inhibition of parasite growth was determined by dye binding assay using SYBR Green (Invitrogen). fluorescence was measuring using the EnVision Multimode Plate Reader (PerkinElmer) with excitation and emission wavelengths of FITC 485/FITC 535. IC_50_ values were calculated using nonlinear regression in GraphPad Prism (log(inhibitor) *versus* normalized response – Variable slope).

For the DCUQ assay, growth of 3D7-WT and *Pf*CLS-HAglmS parasites in the presence of different concentrations (0, 10, 25 μM) of decylubiquinone (DCUQ, Sigma) or DMSO was analysed over two parasite cycles by flow cytometry. As a positive control, WT and *Pf*CLS-HA*glm*S parasites were treated with Atovaquone in addition to DCUQ/DMSO. Culture media containing all drugs and solvent controls were changed regularly until the analysis performed.

### Mitochondrial enrichment

The preparation of mitochondria-enriched fraction was carried out as described earlier with some minor modification (48, 49). Parasites were harvested by using 0.15% saponin treatment from the treated (*Pf*CLS-iKD) and control set. The cell pellet was resuspended in HEPES buffer and lysed using a nitrogen cavitation chamber (#4639 Parr Instrument Company) at a pressure of 1800 to 2000 psi for 20 min on ice. The purified supernatant centrifuged at 22,000×*g* for 30 min at 4 °C and the pellet was resuspended in mitochondria isolation buffer followed by by sucrose density gradient centrifugation, the mitochondrial band was isolated and stored at −80 °C until further analysis.

### High-resolution blue and clear-native PAGE

For blue-native PAGE, mitochondria-enriched fraction was suspended in solubilization buffer (50 mM Bis–Tris–HCl, pH 7.0, 0.5 mM EDTA, 1% (w/v) dodecyl maltoside (DDM)) and combined with the sample buffer containing Coomassie G250 (NativePAGE, Invitrogen). Samples were separated on a NativePAGE 4 to 16% Bis-Tris gel. NativeMark was used as a molecular weight marker.

Clear-native PAGE for complex IV and complex V activity was performed as described previously (49). Briefly, whole parasite samples were suspended in solubilization buffer (50 mM NaCl, 50 mM Imidazole, 2 mM 6-aminohexanoic acid, 1 mM EDTA–HCl pH 7.0, 2% (w/v) n-dodecylmaltoside) and the supernatant containing solubilized membrane proteins was mixed with NativePAGE sample buffer (4 × ) and separated on a NativePAGE 4 to 16% Bis-Tris gel. NativeMark was used as a molecular weight marker.

### In-gel activity of complex IV and complex V

Activity stains were carried out as described previously (49, 95). Briefly, gels were equilibrated in buffer without staining reagents for 10 min. For complex IV, oxidation activity was shown using 50 mM KH_2_PO_4_, pH 7.2, 1 mg ml^−1^ cytochrome *c*, 0.1% (w/v) 3,3′-diaminobenzidine tetrahydrochloride. Stains were visible after 1 to 3 h. Staining was continued with pictures taken of the stained gels at regular intervals.

For complex V, ATP hydrolysis activity was visualized using 35 mM Tris, 270 mM glycine, pH 8.4, 14 mM MgCl_2_, 10 mM ATP, 0.3% (w/v) Pb (NO_3_)_2_. Following incubation, the gel was incubated in 10 ml complex V substrate overnight (∼16 h). Pictures were taken against a black background for optimal visualization of white lead precipitates.

### Metabolite extraction

Metabolites were extracted using a biphasic methanol-chloroform-water extraction protocol as described previously (96). Briefly, synchronized parasite cultures were harvested at the desired time point (30 hpi and 40 hpi) from both sets (control and *Pf*CLS-iKD) by saponin lysis (0.05% in PBS) on ice to remove host erythrocyte material, followed by three washes with ice-cold PBS. The parasite pellets were then flash-frozen in liquid nitrogen and stored at −80 °C until extraction. For extraction, pellets were resuspended in ice-cold 80% methanol, followed by brief sonication of 10 min and then addition of chloroform, water to achieve a final solvent ratio of 4:3:2 (methanol:chloroform:water, v/v/v). The upper aqueous phase was carefully transferred to a glass mass spectrometry insert and dried under vacuum using a SpeedVac concentrator (Thermo Scientific). Samples were resuspended in 20 μl of methoxyamine-HCl (20 mg/ml) prepared in pyridine, sealed and incubated overnight with shaking at ambient temperature. The next day, 20 μl of BSTFA was added to each sample and incubated for 1 h prior to GC-MS analysis.

### Statistical analysis

For statistical analysis of differences between two groups, unpaired two-tailed Student’s *t*-tests were used. For statistical analysis of differences between more than two groups, a one-way analysis of variance, followed by a Holm-Sidak multiple comparison test, was performed. All statistical tests were done in GraphPad Prism V 10.1. *p* values of <0.05 were considered significant. Statistical details (*n* numbers, tests used, definition of the error bars) are described in the figure legends.

## Data availability

All data supporting the findings of this study are included within the manuscript and its supporting information. Source data files containing all numerical values used for GraphPad Prism analyses, including replicate measurements, as well as lipidomic and metabolite quantification datasets, are provided as Supporting Data.

## Supporting information

This article contains supporting information (57, 97).

## Conflict of interest

The authors declare that they have no conflicts of interest with the contents of this article.
