# Supplementary material for: A prokaryotic-like cardiolipin synthase is essential to maintain mitochondrial function, lipid homeostasis, and survival of the blood stage malaria parasite Plasmodium falciparum
Source: J Biol Chem. 2026 May 26;302(7):113188. doi: 10.1016/j.jbc.2026.113188 (PMC13330675; doi:10.1016/j.jbc.2026.113188)
Supplement: Supplementary Data — 1 [file mmc1.docx]

**Supplementary Data**

**Prokaryotic Cardiolipin Synthase in Plasmodium falciparum is essential in maintaining mitochondrial membrane integrity, function and lipid homeostasis**

Md Muzahidul Islam ^a^**^#^**, Md Omair Anwar ^a^**^#^**, Yoshiki Yamaryo-Botté^b^, Pragyan P. Rath^a^,

Madiha Abbas, Vandana Thakur^a^, Sumit Rathore^c^, Cyrille Y. Botté^b*^ and Asif Mohmmed^a*^

**Supplementary data:**

**Phylogenetic analyses reveal that *Pf*CLS is a bacterial type CLS:**

Cardiolipin synthase functions in either CLS_pld pathway or CLS_cap pathway. To determine the evolutionary placement of *Pf*CLS, phylogenetic analyis was performed including various sequences of CLS from different organisms (Appendix 1). Protein sequences were extracted from NCBI or UniProt database and were aligned using ClustalW package in MEGA 11 software. The aligned sequences were subjected to phylogenetic tree construction using the Maximum Likelihood method with 1000 bootstrap in the MEGA 11 software (Tamura, Stecher, & Kumar, 2021). The final figure was prepared through the iTOL server. Our search for a potential cardiolipin synthase in *Plasmodium falciparum* proteome revealed a single protein with two tandem phospholipase D domains (InterPro domain ID IPR001736) (Figure 1A). *Pf*CLS (PF3D7_0609400) is a 603 amino acid protein, where each domain harbours the signature catalytic HKD motif (**H**X**K**XXXX**D**), as shown in Fig. S1B. Domain 1 (182-208 aa) contains **H**_187_R**K**ILIV**D**_194_, while the second domain (478-500 aa) has **H**_478_A**K**NLVV**D**_485_. All *Plasmodium* species have a single representative of this protein and a sequence alignment shows more than 55% identity amongst them. The sequence conservation is stronger within the Apicomplexans, compared to higher eukaryotes. *Pf*CLS shares >20% identity with the human homologue. Our phylogenetic analysis divulged a unique pattern, where CLS from Clade E organisms branches out closer with CLS from bacteria (Fig. S1C). Hence, these protists do not contain the eukaryotic_cap type CLS. Cardiolipin (CL) biosynthesis proceeds through a conserved upstream pathway in both eukaryotic and prokaryotic systems, beginning with the conversion of phosphatidic acid (PA) to CDP-diacylglycerol (CDP-DAG) by CDP-DAG synthase, followed by sequential formation of phosphatidylglycerophosphate (PGP) and its dephosphorylation to phosphatidylglycerol (PG). However, the terminal step of CL synthesis differs between the two systems. In eukaryotes, cardiolipin synthase catalyzes the condensation of PG with CDP-DAG, generating CL with the release of CMP. In contrast, prokaryotic cardiolipin synthase utilizes two phospholipid molecules, typically PG, to produce CL, releasing glycerol as a byproduct (Fig. S1D). Notably, our findings indicate that, although *Plasmodium falciparum* is a eukaryote, *Pf*CLS aligns with the prokaryotic-type pathway, suggesting a mechanistic divergence from the canonical eukaryotic system and that is absent in humans too. The Alphafold structure models were downloaded for all the proteins. *Pf*CLS is Salmon coloured, *Ec*CLSA is Limegreen, *Ec*CLSB is Cyan, *Ec*CLSC is violet, and *Hs*CRLS is Purple in colour. Pymol 3.1.3 was used for visualisation and figure preparation. We superimposed *Pf*CLS onto *E. Coli* and Human structure models. The active sites were compared and analysed. Structural comparisons revealed that Homo sapiens cardiolipin synthase (*Hs*CRLS) is a smaller protein comprising only six α-helices, whereas Plasmodium falciparum CLS (*Pf*CLS) is significantly larger and possesses a distinct active site architecture. Notably, superimposition of the Escherichia coli CLS (*Ec*CLS A) with *Pf*CLS demonstrated a higher degree of structural similarity. Both proteins share comparable overall sizes and exhibit a similar active site conformation. The superimposed structures show a strong alignment of the active site, including the conserved HKD motifs, underscoring the prokaryotic structural ancestry of *Pf*CLS. Regions that do not align between the two proteins are indicated in gray, while the inset highlights the detailed alignment of the active site. Residue numbering is based on the *Pf*CLS sequence (Fig. S2).

**Cloning, expression and purification of recombinant PfCLS protein Phospholipase D (PLD) activity assay and enzyme kinetics**

A DNA fragment corresponding to both Phospholipase domain of *Pf*CLS (amino acid position 61-547) was codon optimized and synthesized from Genscript and cloned in to pET28b vector between *Nco*I and *XhoI* sites to give pET28b- *Pf*CLS -His6 construct (Fig. S3A). The recombinant protein *Pf*CLS was expressed as soluble protein in the cytosol of the *E. coli* Arctic cells (Novogen). For expression, bacterial cells having pET28b- *Pf*CLS -His6 was grown in of LB medium supplemented with kanamycin at 37°C to an absorbance (A600) of ~0.6–0.8. Expression of the fusion proteins were induced using 1mM isopropyl b-D-thiogalactoside (IPTG) overnight at 12°C. Next day cells were harvested and resuspended into resuspension buffer (100mM Tris, pH7.5, 250mM NaCl,1mM DTT) and lysed by sonication (Vibra cell sonicator). The supernatant was subjected a combination of Ni-NTA affinity chromatography followed by size-exclusion chromatography. Eluted fractions were subjected to SDS PAGE and Western blot analysis to assess the purity of the purified recombinant protein.

The corresponding recombinant protein was purified, which migrated on SDS-PAGE at the predicted size of ~54 kDa (Fig. S3B). The purified recombinant *Pf*CLS was used to standardize a fluorescent-based activity assay to quantify its putative Phospholipase D activity, using LPC as a standard substrate (Fig. S3C-E).

For assessing the enzymatic activity of the recombinant *Pf*CLS, a Phospholipase D activity assay was established using lysophosphatidylcholine as substrate as described earlier (Asad et al., 2021).Briefly, the activity assay reaction mixture (200 mL total volume) contained 16:0 lysophosphatidylcholine (LPC) as substrate, 20mg (285pmol) of recombinant protein, 0.1 unit glycerophosphodiesterase (Sigma), 0.2 U/ml choline oxidase (Sigma 26978), 2U/ml horseradish peroxidase (Sigma P8125), 100mM Amplex Red (Invitrogen) in a reaction buffer (50mM Tris pH 8.0, 5mM CaCl2). The *Pf*CLS cleaves the acyl chain from LPC resulting the generation of glycerophosphocholine on which glycerophosphodiesterase acts and cleaves the choline moiety. This choline was oxidized by choline oxidase to produce betaine and H_2_O_2_. Finally, H_2_O_2_ in presence of horseradish peroxidase, reacts with Amplex Red reagent in 1:1 stoichiometry to generate the highly fluorescent product resorufin, which has absorption and fluorescence emission maxima of approximately 571 nM and 585 nM respectively. The PL-D activity was monitored by measuring fluorescence intensities (530 ex/ 590 em) for 6 h using Spectramax M2 microplate reader. The assay was performed by varying the amount of protein and substrate to get optimum PLD activity. The recombinant *Pf*CLS showed concentration dependent PLD activity in the assay (Fig. S3 C, D), confirming the predicted activity of *Pf*CLS, and the presences of Phospholipase D domain. The *Km* and *Vmax* values for *Pf*CLS were found to be 23.39 μM and 30527 nM/min/mg respectively (Fig. S3E). This result confirmed the presences of Phospholipase D domain in *Plasmodium falciparum*.

**Supplementary Table S1:** **List of primers used in the study**

| Primer | Sequence (5`- 3`) |
| --- | --- |
| 1325A | TTCTTTTTATACATGAGCATTG |
| 1326A | AGCTGCAGCAACACCCCACATCATGTTT |
| 1457A | TACGGATACGCATAATCGG |
| gRNA 1651(+) | AGTTATTGTGCATATCATCT |

**Nucleotide sequence selected for generation of gene knock down construct:**

***Pf*CLSgRNA final oligo:**

*Pf*CLSgRNA 1651(+)
5’ TAAGTATATAATATTGAGTTATTGTGCATATCATCTGTTTTAGAGCTAGAA 3’

Reverse complement:

*Pf*CLSgRNA 1651(-)

5’ TTCTAGCTCTAAAAC AGATGATATGCACAATAACTC AATATTATATACTTA 3’

***Pf*CLSgRNA Donor plasmid construct for transfection:**

ATGAGCATTGTTAATAAAGTAAAAGAAGCAATACATAAAAAAGTCATCGATATAAGGAAACGAAGAAAAGGAAGCGAAGA

AAAACAAAAGAAAGACAAAAAAGATGAAATAAATTTTGATGCTTTAGTCGATAAAAATGTAAATGAATTAAATATAGAAA

ATGAAGAAGAAAGAGAGAAAATAAAAGAAAGATGGAAAGTAATATTGAAAAATAATGCTAAGAGATATGGGAAAATATCT

GAAGGAAATAAAATAGAAATATATAATGAAGGTACATTAGCCTTTCGTGATATATTAAATTCCATTAATAAAGGTAAAAG

ACGTGTTTGGTTAGAGTCCTATATTTTTGATGATTCTAAACTTGCAGAAGAAGTAGTTAATAGTTTATGTAAAGCATCGA

AAAGAGGATGTGATGTTATTTTATTAATTGATTATATAGGAAGTTTAAAAATGAAAAATAAATGGGTACAACAATTAAAA

GAACATGGTGTGCATGTTATATTTTTTAATACATTTTTAAATTCGTTTTTTAATATGTTACCTATATTTTTTCGTGATCA

TAGAAAGATATTAATAGTAGATAATACGGCTTACTGTGGTTCAATGAATGTTGCTGAAAATGTATTTCCCAGTGAGATTT

TTCATGAGTATGAGGAAGGGGATGAGAGAGAAGAGGATACCGAATATTCGGAAGAGATAGGAAAAAAAAAAAAAATATAT

AATGATGAGAAAGAAGAAGGAAATAAGAGTAACATGATTAAAAATTTTGATAATGACATTAATAAAGGTAATATTTATGA

TGATTTTAATAAGGACGTAAATAAAAAAAGGAAATGTCTAGAGTATTATGACTTACATATAAAAATAAAAGGTCCAGCTG

TTAAAGATTTAGCTGATGTATTTATTGATTCGTTAAAAATGTCTAAAAGTTTAATAAGTAGAGAACCTATAGAAGAGCAA

AAAAAATATGCAGATGAAAATTCTTGTTATGTTCAAGTATTAGAATCTAATGTTTTAAGAAAGATTAGATCTATTCAATC

AACATTTGATTATATTATAAGAAATGGAGCTACAAATAATATATATATAACAACAAGTTATTTTTTACCACCAGGATTTT

TAAGAAGAGCCTTATTTTCAGCTTTATATAAAGGTGTTAATATATCATTTTTATTTTCAGGTAATTCTGATGTATTTGGT

GATGTTCCAGCTACTTATTATATAATGAAAAAGATATTAAAAAGAATTGATAGGAAAAAGAAGGCTTTATTAGAATATAA

TAATATAATATCAAATTATATAAATTTACATAAAAGTTTTATTAGATATCCTATAATATTTGATAAATATTATAATAATT

ATCAAAAAAAAAAAAAGGAAAGAAAAAATAAAGGATCAATG*gaattc*AATTTTTATTTTTTTCAAAAAAAACATTGTCAT

GCAAAAAATTTAGTAGTAGATAATTTATGGTGTTCTATTGGATCCTATAATTGGGATCGATTTTCATCAAGAAGAAATCT

TGAAGTTATGATATCCATATTTGATAAAAAAATATGTGATAAATTTATACAAGAACATCAAAATAAAATCAGTCATGATT

CTATTCAAATAACATTATCTCAACTAATTAATAGAAATTTTCTTCAAATATTTATGAGTTA**C**TGTGC**T**TATCA**C**CTTGG**T**

AAATTATCGGG**A**AGAAATAT**A**TTTGATGG**T**TTGTCTAA**C**AATAACAA**G**AAAACTAT**T**CTCAGAAAAGC**A**ATCATAAA**A**AA

GTATTT**G**AATGACAA**C**TGTATACA**G**AATATATCTTT**G**AACATGATGTGGGG**A**GTT*ggtacc*TACCCGTACGACGTCCCGG

ACTACGCTGGCTATCCCTATGATGTGCCCGATTATGCGTATCCGTACGATGTTCCAGATTATGCCGCTGCTAGC**TAA**CCA

GGCGCGACGCGTACTAGCTAACATGGCGCGACGCGTACTAGTCAGACCAGCTGTAATTATAGCGCCCGAACTAAGCGCCC

GGAAAAAGGCTTAGTTGACGAGGATGGAGGTTATCGAATTTTCGGCGGATGCCTCCCGGCTGAGTGTGCAGATCACAGCC

GTAAGGATTTCTTCAAACCAAGGGGGTGACTCCTTGAACAAAGAGAAATCACATGATCT*ctcgag*CATGAGTACAAAAAA

AAAAAAAAAAAAAAAAAAAAAAAAAAAAAAAAAAAAAAAAAAAAAAAAAAAAAAAAAAAAAAAAAATAATAAAAAAATAA

TGGGTGGTATGATGCAAAATATATAATACGAAATATGAACTAAGTGGAAAATATAAAATAAAAAATAAAAAATATAAAAT

AAAAAATAAAAAAATAACAAATATATAATAAGAAGTATAATATGGAAGCTATAAAATATATAATGTAAAATATAAAATAT

ATATCGTGAAAATGTCATCACAACATGACATATACATATATATAT*ccatgg*

***Pf*CLS (PF3D7_0609400) Sequence.**

***EcoRI***-HR1-***KpnI***-HA-stop-glmS-***XhoI***-HR2-***NcoI***

Underlined Sequence: recodonized sequence (Changed Nucleotides are marked **red)**

Homology region 1 (HR1): Highlighted in light gray

Homology region 2 (HR2): Highlighted in deep gray

gRNA: Highlighted in purple

HA tag: Highlighted in green

glmS tag: Highlighted in cyan

**References:**

Asad, M., Yamaryo-Botte, Y., Hossain, M. E., Thakur, V., Jain, S., Datta, G., . . . Mohmmed, A. (2021). An essential vesicular-trafficking phospholipase mediates neutral lipid synthesis and contributes to hemozoin formation in Plasmodium falciparum. *BMC Biol, 19*(1), 159. doi:10.1186/s12915-021-01042-z

Tamura, K., Stecher, G., & Kumar, S. (2021). MEGA11: Molecular Evolutionary Genetics Analysis Version 11. *Molecular Biology and Evolution, 38*(7), 3022-3027. doi:10.1093/molbev/msab120

**Supplementary Figures**

**Figure S1**


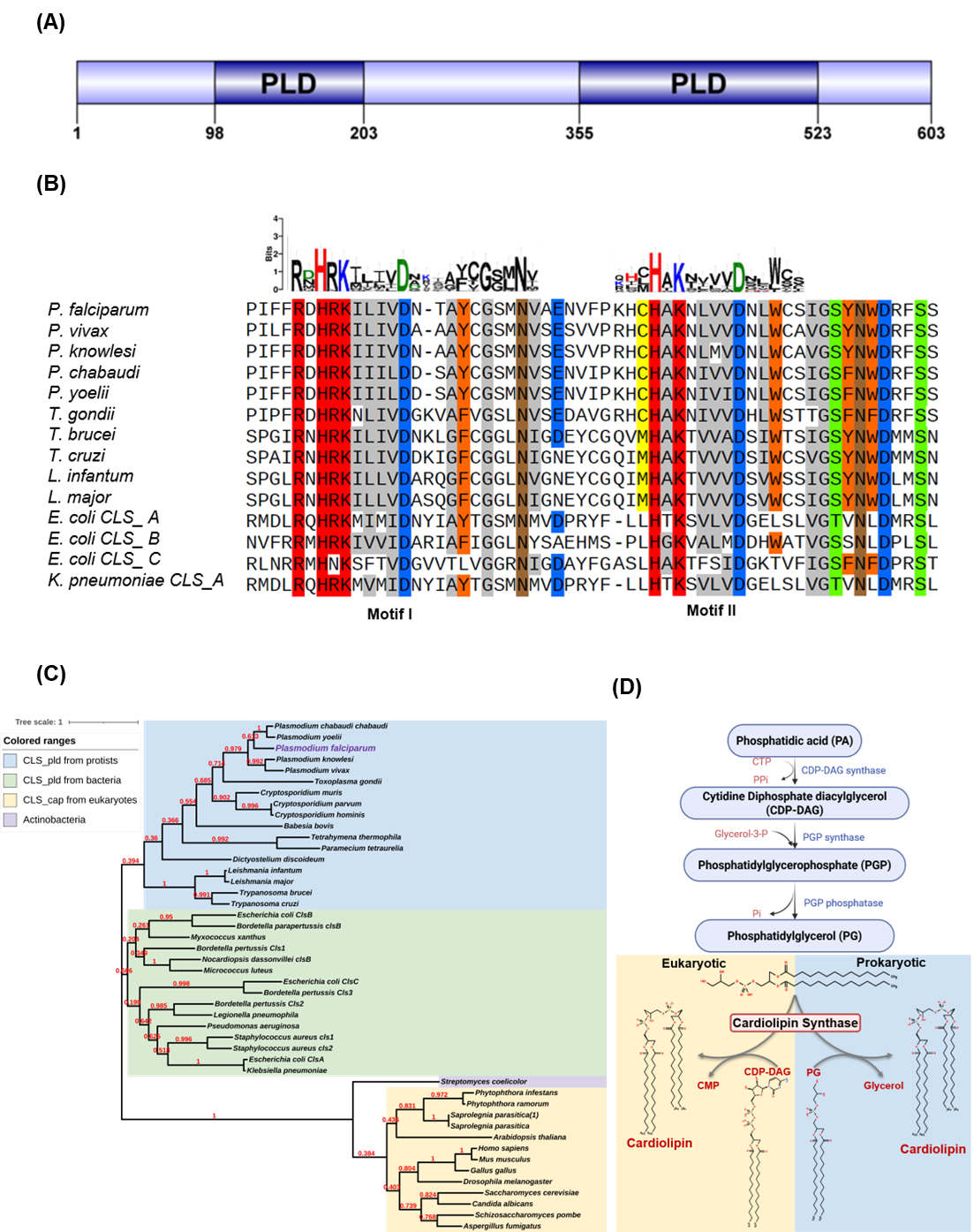


**Figure S1: *Plasmodium falciparum* harbours bacterial type Cardiolipin synthase (CLS) with Phospholipase D domains**

(A) A schematic diagram of the domain organisation in *P. falciparum* CLS (*Pf*CLS), two Phospholipase D domains (PLD1 98-203aa; and PLD2 355-523aa) are marked (dark purple).

(B) Multiple sequence alignment of identified CLS homologues from various apicomplexan parasites and *E. coli*. The figure represents the alignment of both the HKD motifs from all the analysed sequences. On the top of the figure, a weblogo depiction demarcates the HKD motif profile.

(C) Phylogenetic analysis reveals the close homology of apicomplexan PLD domain with that of bacterial type as compared to eukaryotic PLD domain, suggesting presence of presence of bacterial_type_pld domain in *P.falciparum*. Sequence data of organisms from different clades, including fungi, plant, animal, apicomplexan, actinobacteria etc. was analysed. Different coloured ranges are marked in the tree for clear understanding of different type of CLS**.**

(D) Comparison of eukaryotic and prokaryotic cardiolipin biosynthesis pathways. Phosphatidic acid (PA) is converted to CDP-diacylglycerol (CDP-DAG) by CDP-DAG synthase, followed by formation of phosphatidylglycerol (PG) via phosphatidylglycerophosphate (PGP). Cardiolipin (CL) synthesis diverges at the final step: eukaryotic CLS uses PG and CDP-DAG to produce CL with release of CMP, whereas prokaryotic CLS utilizes two PG, releasing glycerol. *Plasmodium falciparum* follows the prokaryotic-type pathway.

# **Figure S2**

**
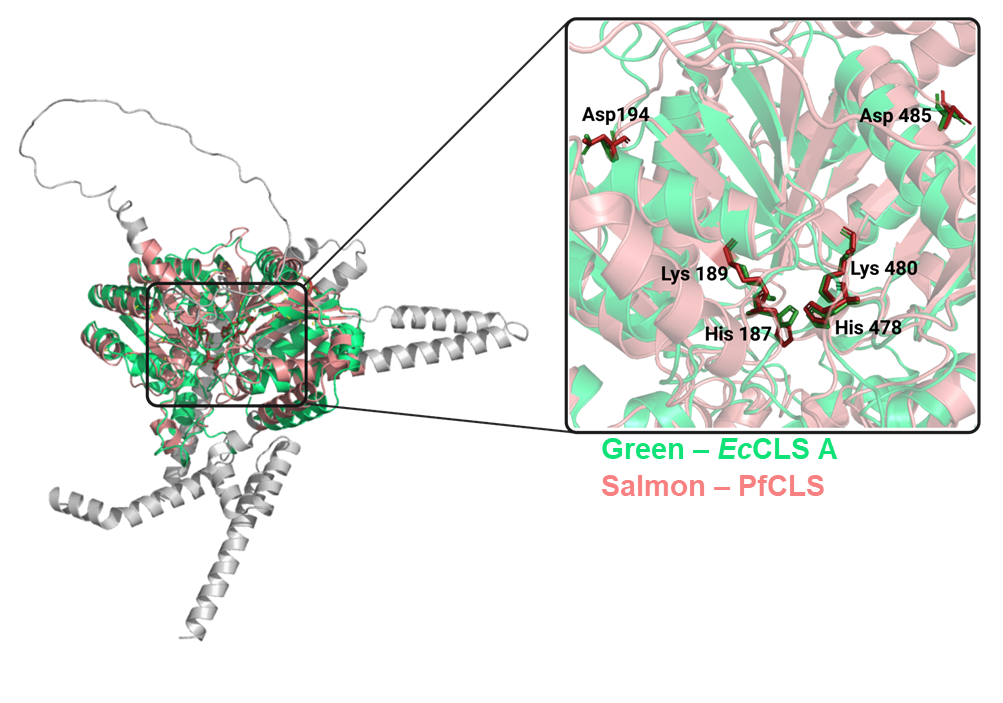
**

**Figure S2: Structural superimposition *Pf*CLS and *E. coli* CLSA**

The superimposed structures show a strong alignment of the active site, including the conserved HKD motifs, underscoring the prokaryotic structural ancestry of *Pf*CLS. Regions that do not align between the two proteins are indicated in gray, while the inset highlights the detailed alignment of the active site. Residue numbering is based on the *Pf*CLS sequence. The insert displays active site residues.

# **Figure S3**


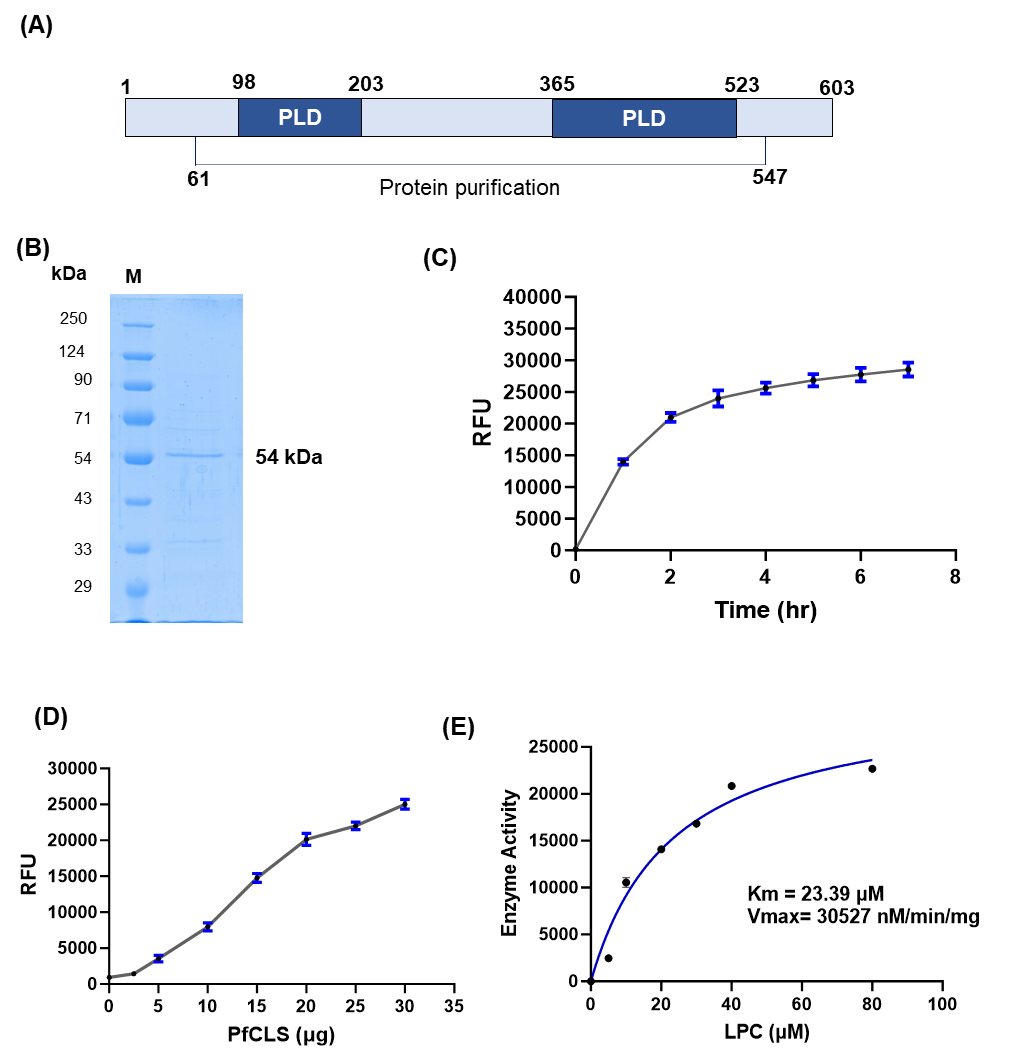


**Figure S3: Biochemical characterization of recombinant *Pf*CLS**

(A) A schematic diagram of the domain organisation in *P. falciparum* CLS (*Pf*CLS), two Phospholipase D domains and regions selected for recombinant protein purification (amino acid position 61-547) has been marked.

(B) The full gene *pfcls* was cloned into pET28b vector and recombinant protein was expressed in BL21 (DE3) *E. coli* cells*.* It was purified by affinity chromatography using Ni^2+-^NTA. Coomassie stained SDS-PAGE showing purified recombinant protein (~54kDa).

(C-E) *In vitro* activity of recombinant *Pf*CLS was assessed by Phospholipase D assay using LPC as substrate and Amplex-Red detection kit. (C) Time dependent *Pf*CLS activity using 25μg of recombinant protein in the assay reaction. (D) PLD activity of *Pf*CLS at different protein concentrations (2.5 – 30 μg) at 6h time point. (E) Line graph showing Michaelis-Menten fit curve developed for *Pf*CLS using different concentrations of LPC substrate. The *K*m and *V*max values of *Pf*CLS were found to be 20.80μM and 27441nM/min/mg respectively.

# **Figure S4**

**
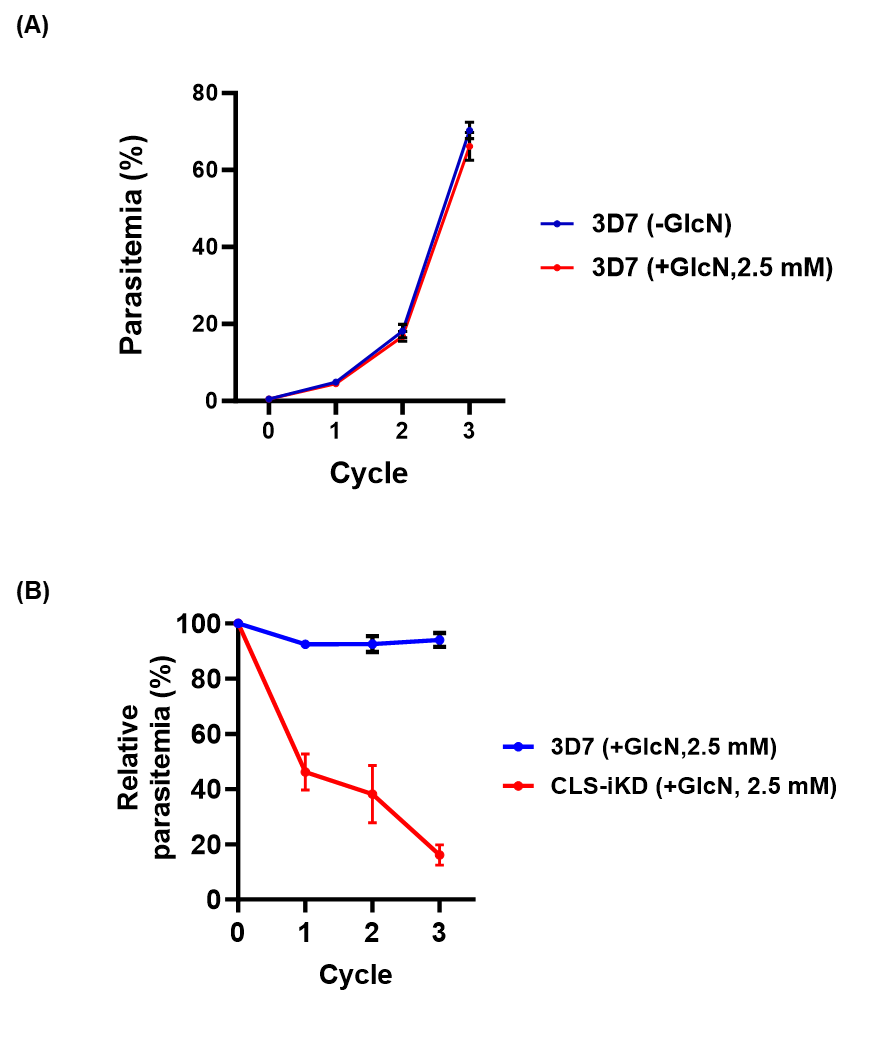
**

**Figure S4: Effect of Glucosamine on the growth of the wild-type parasite line (3D7).**

(A) Graph showing growth of wild-type parasites (3D7) in the presence of glucosamine (2.5mM) in comparison to control (0 mM), over three asexual cycles; growth was determined by counting total parasitemia at 48, 96, 144 and 192 hpi. All analyses were carried out in triplicate and error bars represent the standard deviation.

(B) Relative parasitemia of *Pf*CLS-iKD parasites compared to 3D7 in the presence of 2.5 mM GlcN. All analyses were performed in triplicate (n=3); error bars indicate standard deviations.

**
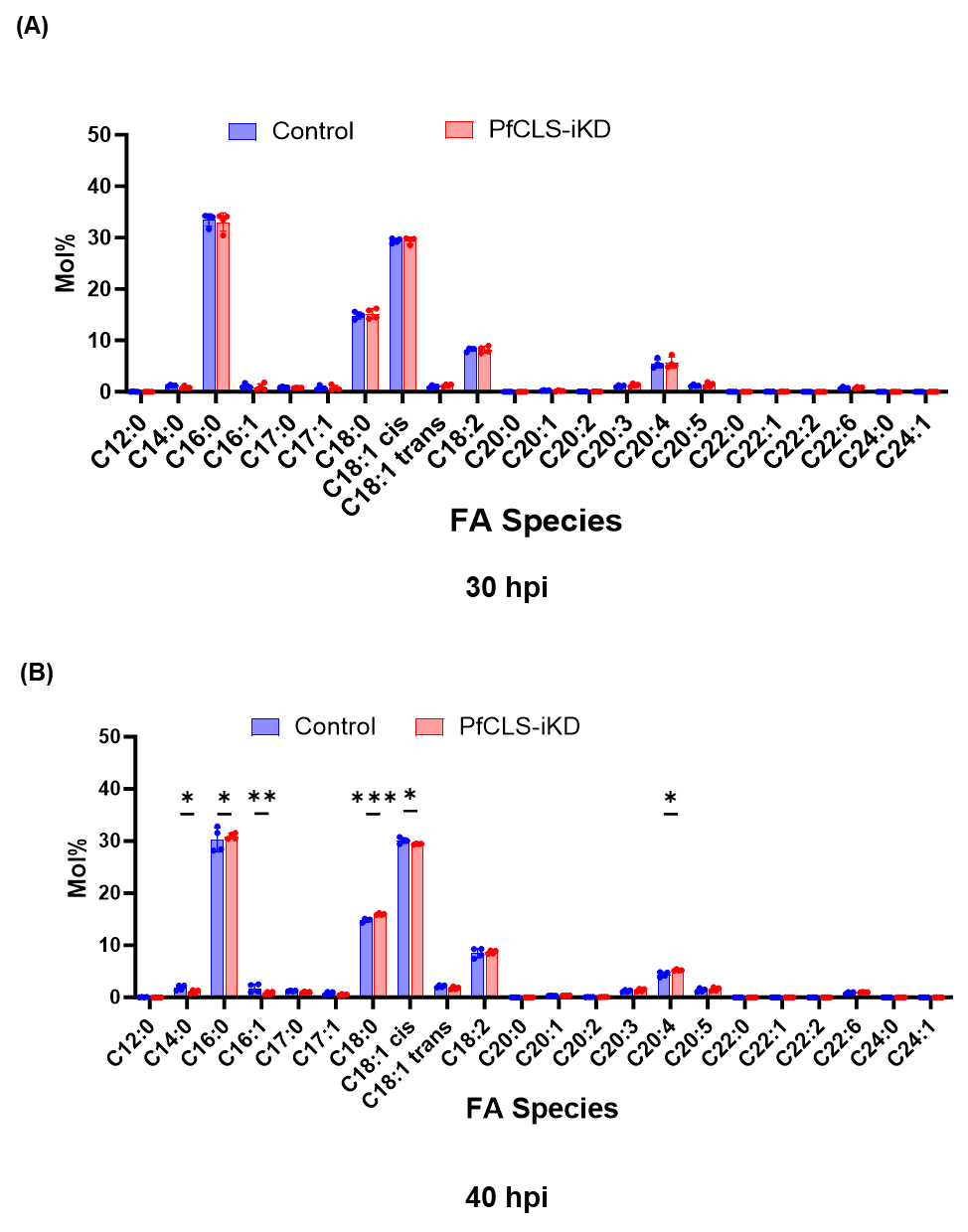
Figure S5**

**Figure S5: Alteration in lipid composition by inducible knock-down of *Pf*CLS in the parasites**

(A–B) Graphs showing the composition (Mol%) of different classes of fatty acids in control and *Pf*CLS-iKD parasites at 30 hpi and 40 hpi. No significant differences were observed at 30 hpi, whereas at 40 hpi, significant changes were detected in selected fatty acids (C14:0, C16:0, C18:0, and C18:1) between control and *Pf*CLS-iKD parasites. Data represent mean ± SD from at least three independent experiments (n ≥ 3). Statistical significance was assessed using an unpaired two-tailed Student’s t-test.

**Figure S6**

**
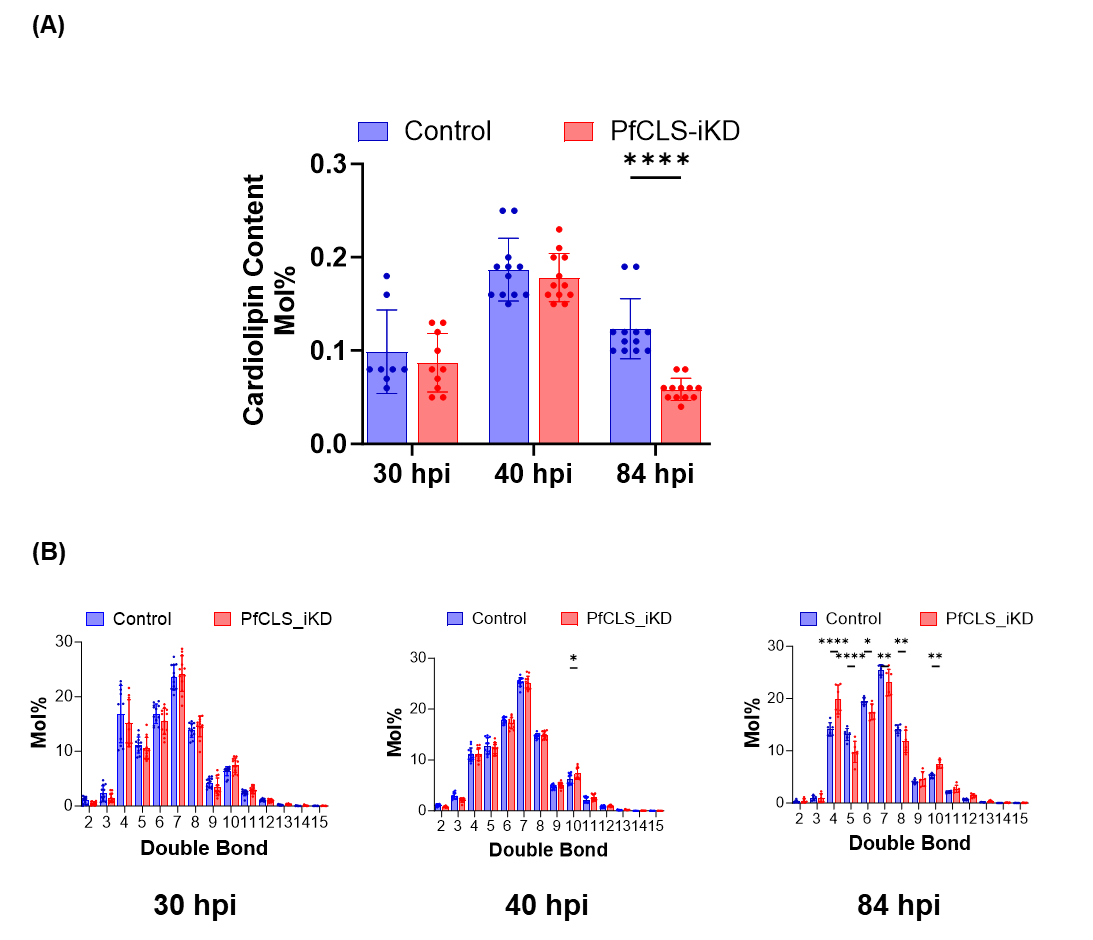
**

**Figure S6: Alteration in cardiolipin (CL) content in control and *Pf*CLS-iKD parasites**

(A) Quantification of cardiolipin (CL) content (mol%) in control and *Pf*CLS-iKD parasites at 30 hpi, 40 hpi, and 84 hpi, showing a significant reduction in CL levels in *Pf*CLS-iKD parasites at 84 hpi. Data represent mean ± SD from at least three independent experiments (n ≥ 3). Statistical significance was determined using an unpaired two-tailed Student’s t-test.

(B) Distribution of double bonds in CL species in control and *Pf*CLS-iKD parasites at 30 hpi, 40 hpi, and 84 hpi. Data are presented as mean ± SD from at least three independent experiments (n ≥ 3). Statistical significance is indicated as follows: *p < 0.05, **p < 0.01, ***p < 0.001, ****p < 0.0001.

**Figure S7**

**Figure S7:** **Fatty acid profile for Cardiolipin showing composition of molecular species.**

Profile of abundance of fatty acid species in control and *Pf*CLS-iKD sets for CL at 30 hpi, 40 hpi and 84 hpi. 40 hpi and 84 hpi have altered FA abundance and combination in the *Pf*CLS-iKD set in comparison to the control, indicating overall disruption in cardiolipin homeostasis. All analyses were performed in triplicate (*n* = 3) or more; the error bars indicate standard deviations. **** signifies *p*<0.0001, while *** signifies *p*<0.001, ** signifies *p*<0.01 and * signifies *p*<0.05. The *p* values were determined by unpaired Student’s *t*-test.

**Figure S8**

**
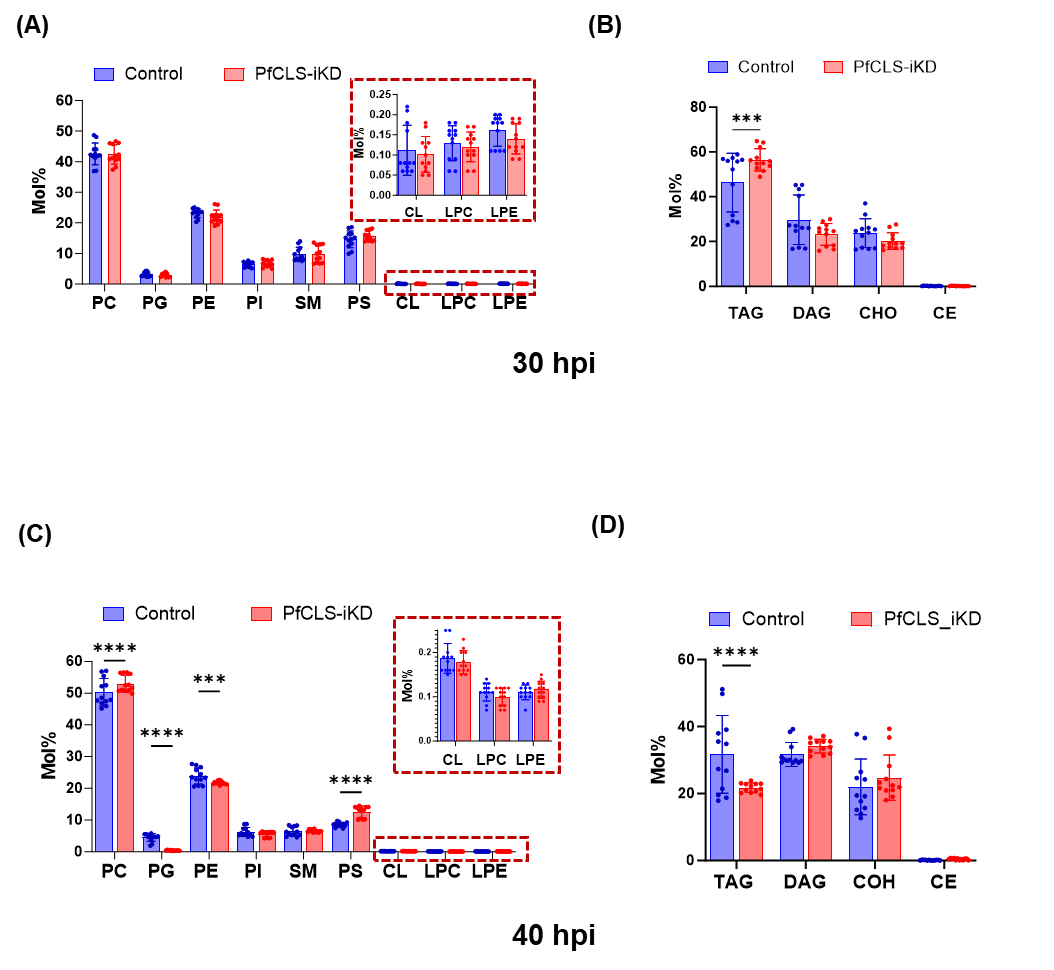
Figure S8:** **Alteration in membrane phospholipid composition by inducible knock-down of *Pf*CLS in the parasites**

(A and C) Graphs showing composition (Mol%) of major phospholipid species (PLs) in control and *Pf*CLS-iKD parasites at 30 hpi (A) and 40 hpi (C), showing no significant changes in fatty acid composition between control and *Pf*CLS-iKD parasites. Insets show enlarged sections highlighting the relative abundance of CL, LPC, and LPE in *Pf*CLS-iKD parasites compared to control.

(B and D) Analysis of neutral lipids levels (in Mol%) in control and *Pf*CLS-iKD parasites at 30 hpi (B) and 40 hpi (D).

Statistical significance for each lipid class in all panels (A–D) was assessed using an **unpaired two-tailed Student’s t-test,** with **Holm–Šídák correction for multiple comparisons.** All data are presented as mean ± SD from at least three independent experiments (n ≥ 3). Statistical significance is indicated as follows: **p < 0.01, **p < 0.005, ***p < 0.001*. Abbreviations: PE, phosphatidylethanolamine; PC, phosphatidylcholine; PS, phosphatidylserine; PI, phosphatidylinositol; SM, sphingomyelin; CL, cardiolipin; LPI, lysophosphatidylinositol; PG, phosphatidylglycerol.

**Figure S9 A**

**
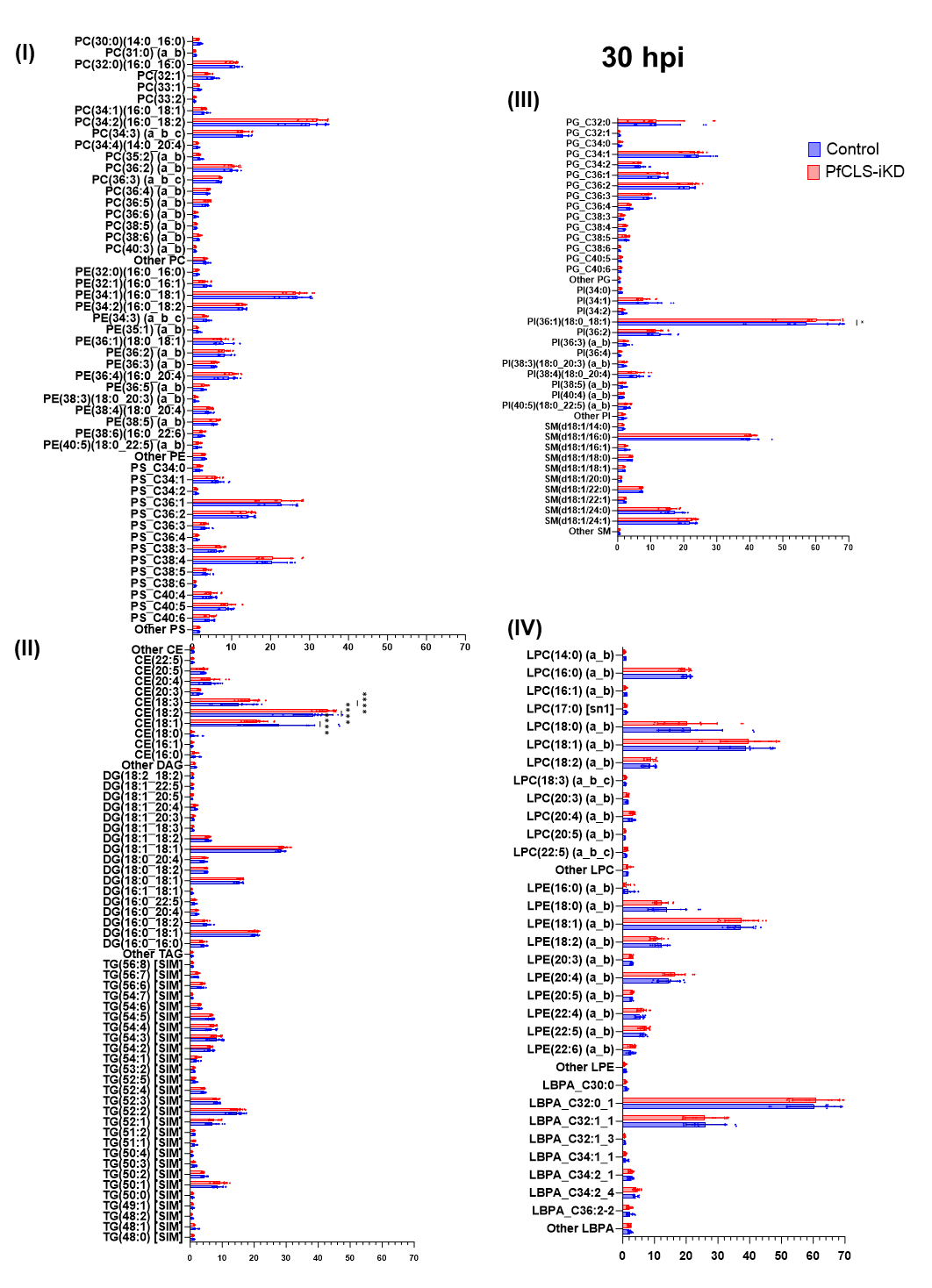
**

**Figure S9 B**

**
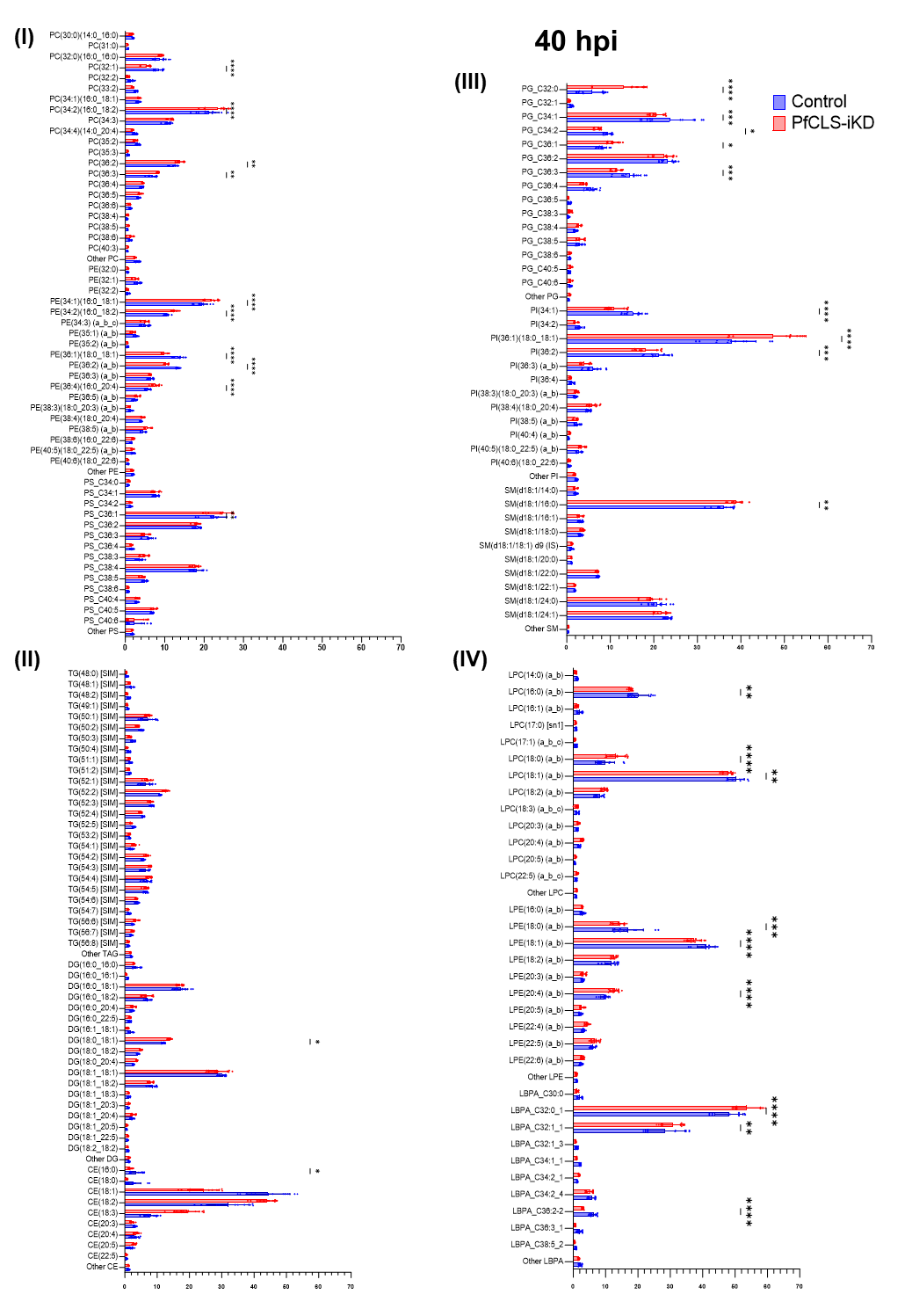
**

**Figure S9A and S9B: FA profile for different phospholipid showing relative abundance of individual molecular species.**

(A-B) Profile of abundance of fatty acid species in control and *Pf*CLS-iKD sets for other major phospholipids (PC, PE, PS, PG, PI, LPE. LPC, LPA, LBPA, SA, CE, TAG and DAG) at 30 hpi (A) and 40 hpi (B). Altered FA abundance and combination of different lipid classes in the *Pf*CLS-iKD set in compare to the control set has been observed and data indicating overall disruption in lipid homeostasis.

Statistical significance for each lipid class in all panels (A–D) was assessed using an unpaired two-tailed Student’s t-test. All data are presented as mean ± SD from at least three independent experiments (n ≥ 3). Statistical significance is indicated as follows: *p < 0.01, **p < 0.005, ***p < 0.001. Abbreviations: PS, phosphatidylserine; PI, phosphatidylinositol; PC, phosphatidylcholine; PG, phosphatidylglycerol; PE, phosphatidylethanolamine; SM, sphingomyelin; CE ceramide; TAG, triacylglycerols; DAG, diacylglycerol; LPE, lysophosphosphatidylethanolamine; LPC, lysophosphatidylcholin, LBPA, lysobismonoacylglycerolphosphate.

**Figure S10**


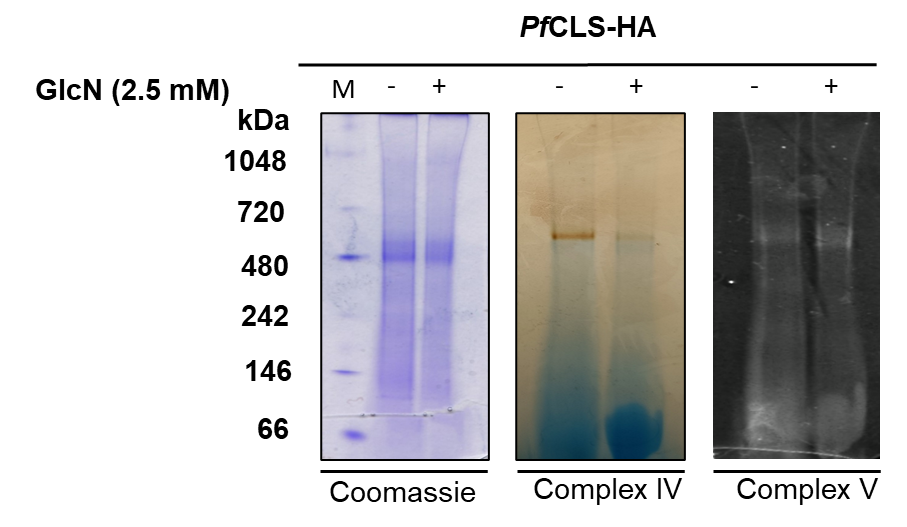


**Figure S10: Depletion of *Pf*CLS hinders mtETC super complex formation.**

Downregulation of *Pf*CLS leads impairs in respiratory complex IV but not in complex V. Mitochondrial enriched fraction from *Pf*CLS-iKD set and control set at 38-40 hpi were separated by high resolution clear-native PAGE, and stained either for complex IV or complex V activity, a parallel gel was stained with Coomassie blue as loading control. Data represent results from the repeat experiment shown in the Figure:7F.
